# Supplementary material for: Do we advise as one likes? The alignment bias in social advice giving
Source: PLoS Comput Biol. 2025 Dec 2;21(12):e1013732. doi: 10.1371/journal.pcbi.1013732 (PMC12688123; doi:10.1371/journal.pcbi.1013732)
Supplement: S1 Text — (DOCX) [file pcbi.1013732.s001.docx]

**S1 Text. Pretests on the stimuli.**

A separate sample of 30 participants (mean age: 21.48 years, range: 20-29 years; 12 males, 18 females) who were distinct from the subject sample recruited for Study 1-4, rated their confidence in price judgements and provide familiarity ratings (ranging from 1 to 11) on the stimuli that we used in the present study.

The descriptive data for these measurements were as follows:

| **Measurement** | | **Condition** | | **Mean** | | ***SD*** | |
| --- | --- | --- | --- | --- | --- | --- | --- |
| Judgement confidence | | Congruent | | 2.7432 | | 0.51911 | |
|  |  | Incongruent | | 2.7593 | | 0.56101 | |
|  |  | Masked | | 2.6972 | | 0.58749 | |
|  |  |  |  |  |  |  |  |
| Familiarity | | Congruent | | 4.416 | | 0.41596 | |
|  |  | Incongruent | | 4.3543 | | 0.53908 | |
|  |  | Masked | | 4.55 | | 0.42652 | |
