# Supplementary material for: Do we advise as one likes? The alignment bias in social advice giving
Source: PLoS Comput Biol. 2025 Dec 2;21(12):e1013732. doi: 10.1371/journal.pcbi.1013732 (PMC12688123; doi:10.1371/journal.pcbi.1013732)
Supplement: S2 Text — (DOCX) [file pcbi.1013732.s002.docx]

**S2 Text. Mixed-effect models specifications.**

| 2.2.1 Advisors aligned their advice towards advisees’ opinions |
| --- |
| Judgement congruency ~ Session (1 vs. 2) |
| Opinion discrepancy ~ Session (1 vs. 2) |
| Investment magnitude ~ Session (1 vs. 2) × Advisee’s risk preference (risk-avoiding vs. risk-seeking) |
| 2.2.2 Advisors exhibited an unselective inclination to re-align with advisees’ opinions |
| Judgement switch ~ Baseline congruency (congruent vs. incongruent) × Advisee’s accuracy (correct vs. incorrect) |
| Judgement switch ~ Baseline congruency (congruent vs. incongruent) × Advisee’s confidence + Baseline confidence |
| OSR ~ Baseline congruency (congruent vs. incongruent) × Advisee’s accuracy (correct vs. incorrect) |
| OSR ~ Baseline congruency (congruent vs. incongruent) × Advisee’s accuracy (correct vs. incorrect) + Baseline opinion discrepancy |
| 3.2.2 The conformity to advisees’ opinions reflects a social basis |
| Judgement congruency ~ Session (2 vs. 3) × Advisee condition (same vs. new) |
| Judgement re-alignment maintenance (in Session 3) ~ Advisee condition (same vs. new) |
| Self-judgement consistency (across Session 2 and 3) ~ Advisee condition (same vs. new) |
| 4.2.1 Model-free analyses |
| Judgement congruency (in Session 2) ~ Preference (neutral vs. aligned vs. misaligned) × Order (neutral-aligned-misaligned vs. neutral-misaligned-aligned) |
| Judgement congruency (in Session 2) ~ Preference (aligned vs. misaligned) |
| Judgement congruency (in Session 2) ~ Trial |
| 5.2 Results |
| Attention check failure ~ Study (3 vs. 4) |
| Investment magnitude ~ Session (1 vs. 2) × Study (3 vs. 4) |
| Judgement congruency (Session 2) ~ Study (3 vs. 4) |

*Note.* All linear mixed-effects models reported in this table included random intercepts for both stimulus and participant, specified as + (1 | stimulus) + (1 | participant). For models that appeared multiple times in the main text, we only indicated the result section of their first occurrence here.
