# Supplementary material for: Do we advise as one likes? The alignment bias in social advice giving
Source: PLoS Comput Biol. 2025 Dec 2;21(12):e1013732. doi: 10.1371/journal.pcbi.1013732 (PMC12688123; doi:10.1371/journal.pcbi.1013732)
Supplement: S3 Text — (DOCX) [file pcbi.1013732.s003.docx]

**S3 Text. Model estimation, model selection, parameter recovery and model recovery.**

*Hierarchical Bayesian model estimation*

We conducted model estimations with hierarchical Bayesian analysis (HBA)[1] using Stan [2] in R. Stan utilizes a Hamiltonian Monte Carlo (HMC; an efficient Markov Chain Monte Carlo, MCMC) sampling scheme to perform full Bayesian inference and obtain the actual posterior distribution. We performed HBA rather than maximum likelihood estimation (MLE) because HBA provides much more stable and accurate estimates than MLE [1]. Following the approach in the “hBayesDM” package [3] for using Stan in the field of reinforcement learning, we assumed, for instance, that a generic individual-level parameter ϕ was drawn from a group-level normal distribution, namely, ϕ ~ Normal (μ_ϕ_, σ_ϕ_), with μ_ϕ_ and σ_ϕ_ being the group-level mean and standard deviation, respectively. Both these group-level parameters were specified with weakly informative priors[1]: μ_ϕ_ ~ Normal (0, 1) and σ_ϕ_ ~ Cauchy (0, 2). This was to ensure that the MCMC sampler traveled over a sufficiently wide range to sample the entire parameter space. In HBA, all group-level parameters and individual-level parameters were simultaneously estimated through the Bayes’ rule by incorporating behavioral data. We fit each candidate model with four independent MCMC chains using 2,000 iterations after 1000 iterations for the initial algorithm warmup per chain, which resulted in 4,000 (= (2000-1000)*4) valid posterior samples. The convergence of MCMC chains was assessed both visually (from the trace plot) and through the Gelman-Rubin R̂ Statistics [4]. R̂ values of all parameters were close to 1.0 (smaller than 1.1 at most in the current study) as below, which indicated adequate convergence.

|  | $w_{s}$ | $w_{ns}$ | $a_{al}^{ac}$ | $a_{al}^{re}$ | $a_{mis}^{ac}$ | $a_{mis}^{re}$ |
| --- | --- | --- | --- | --- | --- | --- |
| Study 3 | 1.009 | 1.011 | 1.012 | 1.008 | 1.010 | 1.015 |
| Study 4 | 1.010 | 1.006 | 1.007 | 1.011 | 1.014 | 1.003 |

*Note.* R̂ values of all parameters in the winning model were close to 1.0 (smaller than 1.1 at most in the current study), which indicated adequate convergence.

*Model selection and posterior predictive check*

For model comparison and model selection, we computed the Leave-One-Out information criterion (LOOIC) score per candidate model[5]. The LOOIC score provides the point-wise estimate (using the entire posterior distribution) of out-of-sample predictive accuracy in a fully Bayesian way, which is more reliable compared to information criteria using point-estimate (e.g., Akaike information criterion, AIC; deviance information criterion, DIC). By convention, a lower LOOIC score indicates better out-of-sample prediction accuracy of the candidate model. We selected the model with the lowest LOOIC as the winning model.

Moreover, given that model comparison provided merely relative performance among candidate models[6,7], we then tested how well our winning model’s posterior prediction was able to capture the key features of the observed data (a.k.a., posterior predictive checks, PPCs). We conducted PPC at three scales [7]: the trial-wise scale, the individual-wise scale, and the grand average scale. Specifically, in trial-wise scale and in individual-wise scale, we conducted correlation tests to test the associations between the synthetic choice data generated by the winning model (with the individual-level parameters acquired from the model estimation) and the true data. In the overall level, we compared the posterior sample distributions generated from the winning model (also, with the individual-level parameters acquired from the model estimation) with the true data, to check if the true data fall within the 95% Highest Density Intervals (HDIs) of the posterior sample distributions.

*Parameter recovery*

Considering that there were multiple free parameters in the winning model (M7 for Study 4), we verified whether parameters were identifiable using parameter recovery. Denoting as a generic parameter for a model, we iterated the following steps:

1. We randomly drew a set of group-level parameters from the parameter space of the winning model. That is, a group-level mean () and a group-level standard deviation () of the parameter . We repeated this procedure for all group-level parameters of the winning model.
2. Next, we simulated 80 synthetic participants, whose parameters were randomly drawn from this set of group-level parameters. That is, individual-level parameters for 80 synthetic participants were sampled from the corresponding group-level parameters (, ) with a normal distribution. We repeated this procedure for all individual-level parameters of the winning model:

1. Then, we used the winning model as a generative tool to simulate behavioral data for our social advice-giving task (Session 2), namely, to simulate their advised judgment options for 66 trials (identical to our experimental task) per participant. Individuals’ judgement opinion (denoted as $\hat{D}_{i}$) were sampled from the sampling distribution conditional on individual-level parameters () from the previous step (i.e., likelihood function):
2. We fit the winning model to the simulated data ($\hat{D}_{i}$) in the same way as we did for the real data (D). That is, we fit the winning model to the simulated individual data ($\hat{D}_{i}$) with MCMC using Stan, and obtained parameter estimates (i.e., posterior distributions) at both the group-level (e.g.,, ) and the individual level (e.g., ).
3. Finally, we compared whether the posterior distributions at both the group-level and the individual-level given the simulated data recovered the actual data generating parameters that were used to simulate those data, sampled from steps (a) and (b)

*Model recovery*

We further conducted a full model-recovery test across the candidate models. This analysis aimed to clarify whether the best-fitting model is uniquely capable of explaining the data or whether alternative models could generate similar behavioral patterns. To this end, we used each candidate model to simulate 10 behavioral datasets (each dataset including 30 participants, each participant consisted of 66 trials) following the identical procedure as we conducted parameter recovery. Then, we refitted all candidate models to these simulated datasets and performed model selection procedure as we did for the real data. Successful recovery would be indicated if the model used to generate the data was also identified as the best-fitting model consistently.


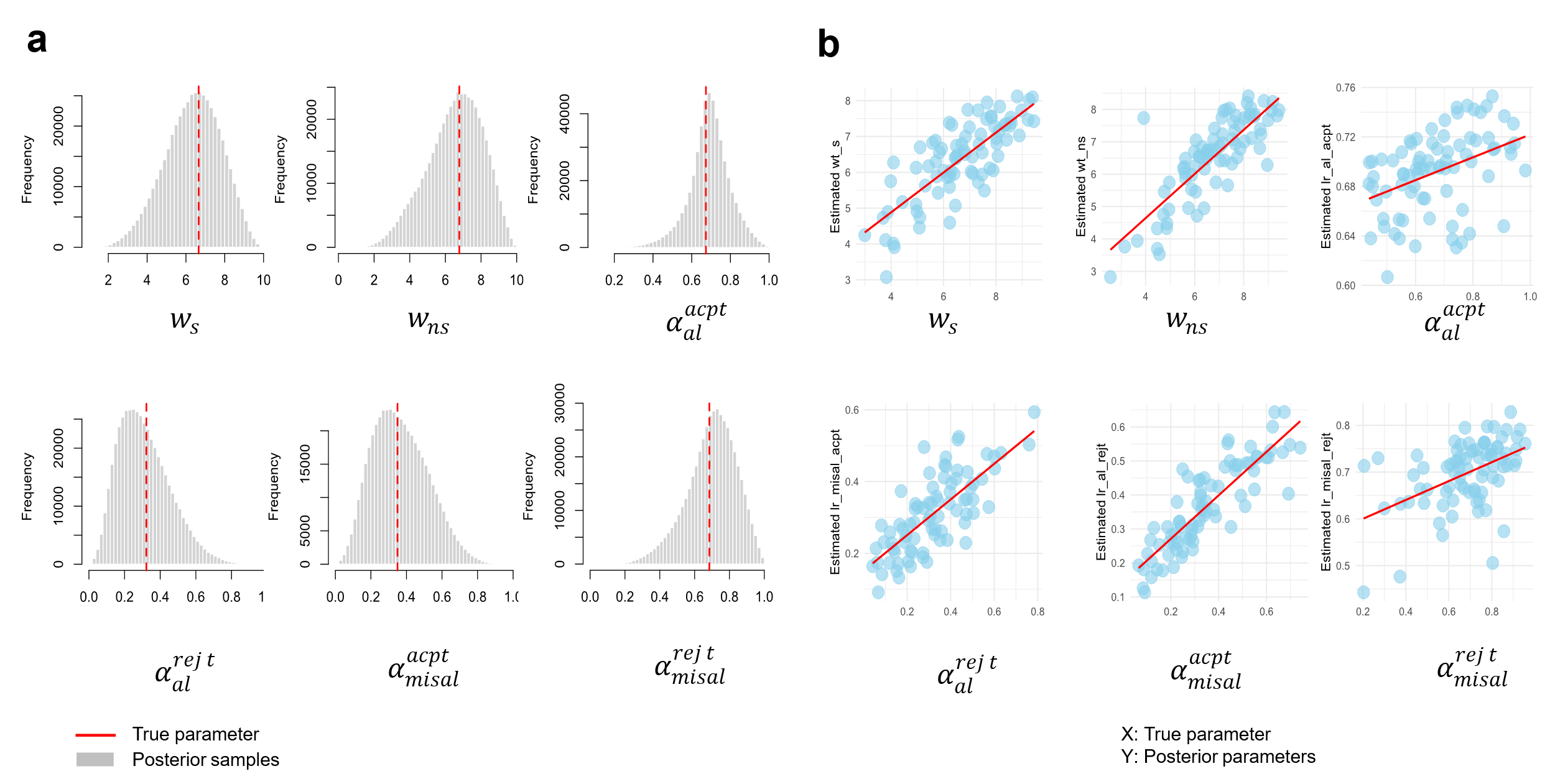


**Fig A.** **Parameter recovery of the winning model.** All parameters could be accurately and selectively recovered, showing proper identifiability of model parameters: (a) for the group-level, “true” parameters (in red) falling within 95% HDI of each parameter’s posterior density; (b) for the individual level, “true” parameters (x-axis) and estimated parameters (y-axis) were well correlated.


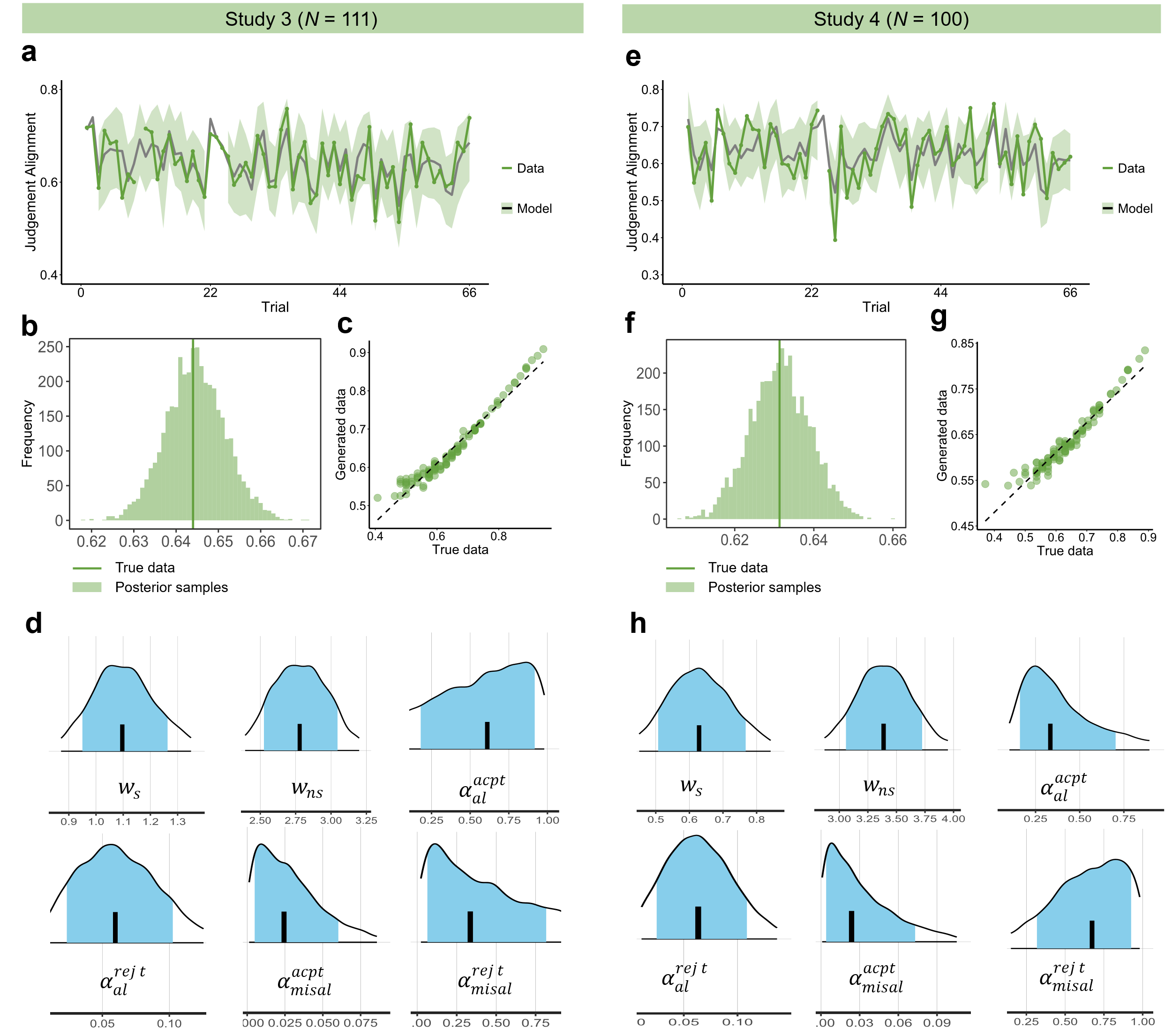


**Fig B.** **Posterior predictive check of the winning model and the posterior distributions of all estimated parameters.** (a) and (e) Trial-wise posterior predicative check. The actual trial-by-trial data well correlated by the predicted data. (b) and (f) Grand-wise posterior predicative check. The true data (dark green vertical line) fall within the 95% Highest Density Intervals (HDIs) of each posterior density (in light green). (c) and (g) Individual-wise posterior predicative check. The actual individual data well correlated by the predicted data. (d) and (h) The posterior distributions of the estimated parameter in the winning model. The vertical line indicates the posterior median, and the blue shading represents the 95% HDI.


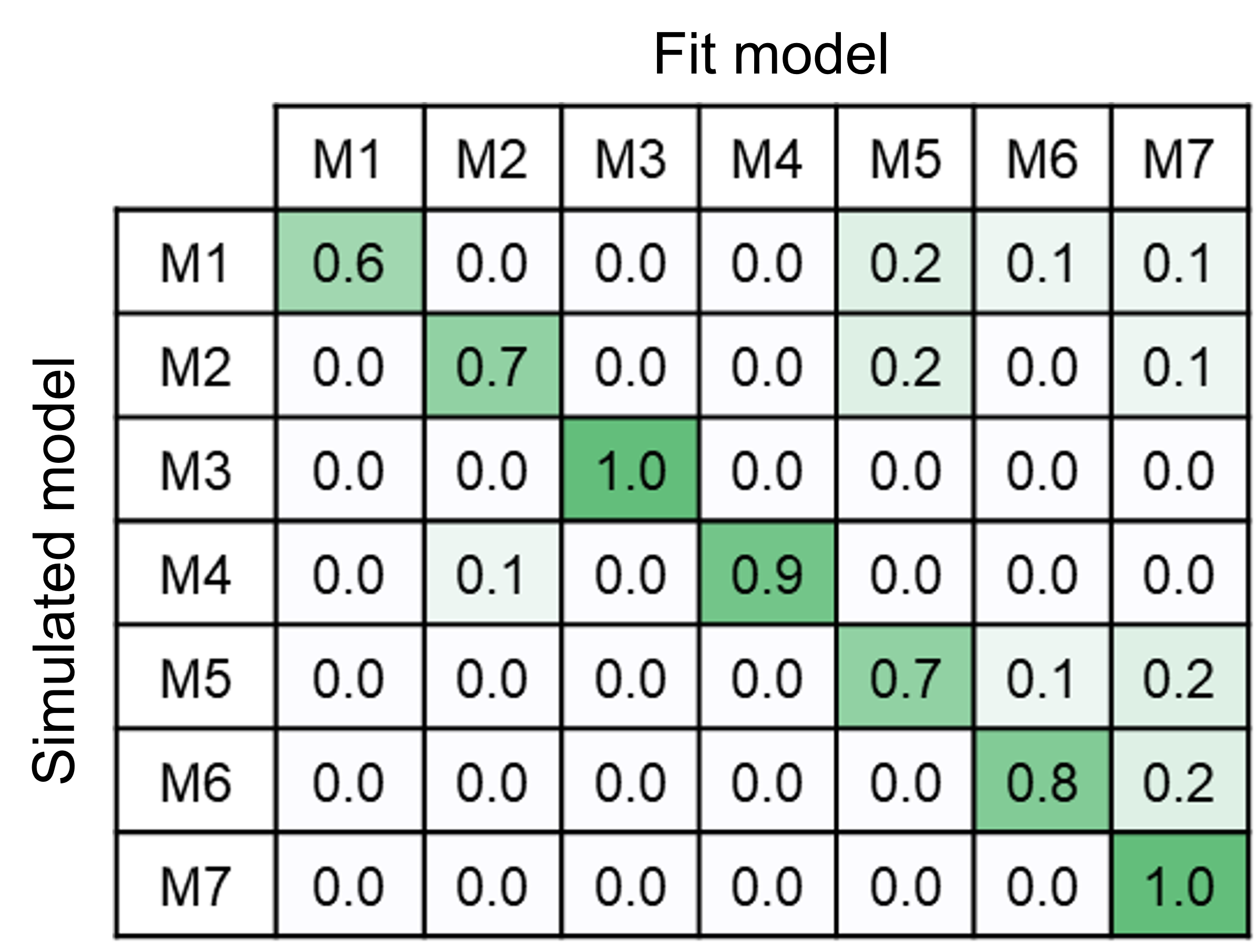


**Fig C.** **Model recovery of all candidate models.** Confusion matrix: *p*(fit model | simulated model) was used to illustrate the performance of model recovery. The larger value in the diagonal indicates better recovery accuracy, whereas off-diagonal values reflect potential model confusion.

**References**

[1] Gelman A, Carlin J, Stern H, et al. Chapman & Hall/CRC texts in statistical science. Bayesian Data Analysis. 2013;

[2] Carpenter B, Gelman A, Hoffman MD, et al. Stan: A Probabilistic Programming Language. J Stat Softw. 2017;76:1.

[3] Ahn W-Y, Haines N, Zhang L. Revealing Neurocomputational Mechanisms of Reinforcement Learning and Decision-Making With the hBayesDM Package. Comput Psychiatr. 2017;1:24–57.

[4] Gelman A, Rubin DB. Inference from Iterative Simulation Using Multiple Sequences. Statistical Science. 1992;7(4):457–472.

[5] Vehtari A, Gelman A, Gabry J. Practical Bayesian model evaluation using leave-one-out cross-validation and WAIC. Stat Comput. 2017;27(5):1413–1432.

[6] Palminteri S, Wyart V, Koechlin E. The Importance of Falsification in Computational Cognitive Modeling. Trends in Cognitive Sciences. 2017;21(6):425–433.

[7] Zhang L, Lengersdorff L, Mikus N, et al. Using reinforcement learning models in social neuroscience: frameworks, pitfalls and suggestions of best practices. Social Cognitive and Affective Neuroscience. 2020;15(6):695–707.
