# Supplementary figures and images for: Do we advise as one likes? The alignment bias in social advice giving
Source: PLoS Comput Biol. 2025 Dec 2;21(12):e1013732. doi: 10.1371/journal.pcbi.1013732 (PMC12688123; doi:10.1371/journal.pcbi.1013732)

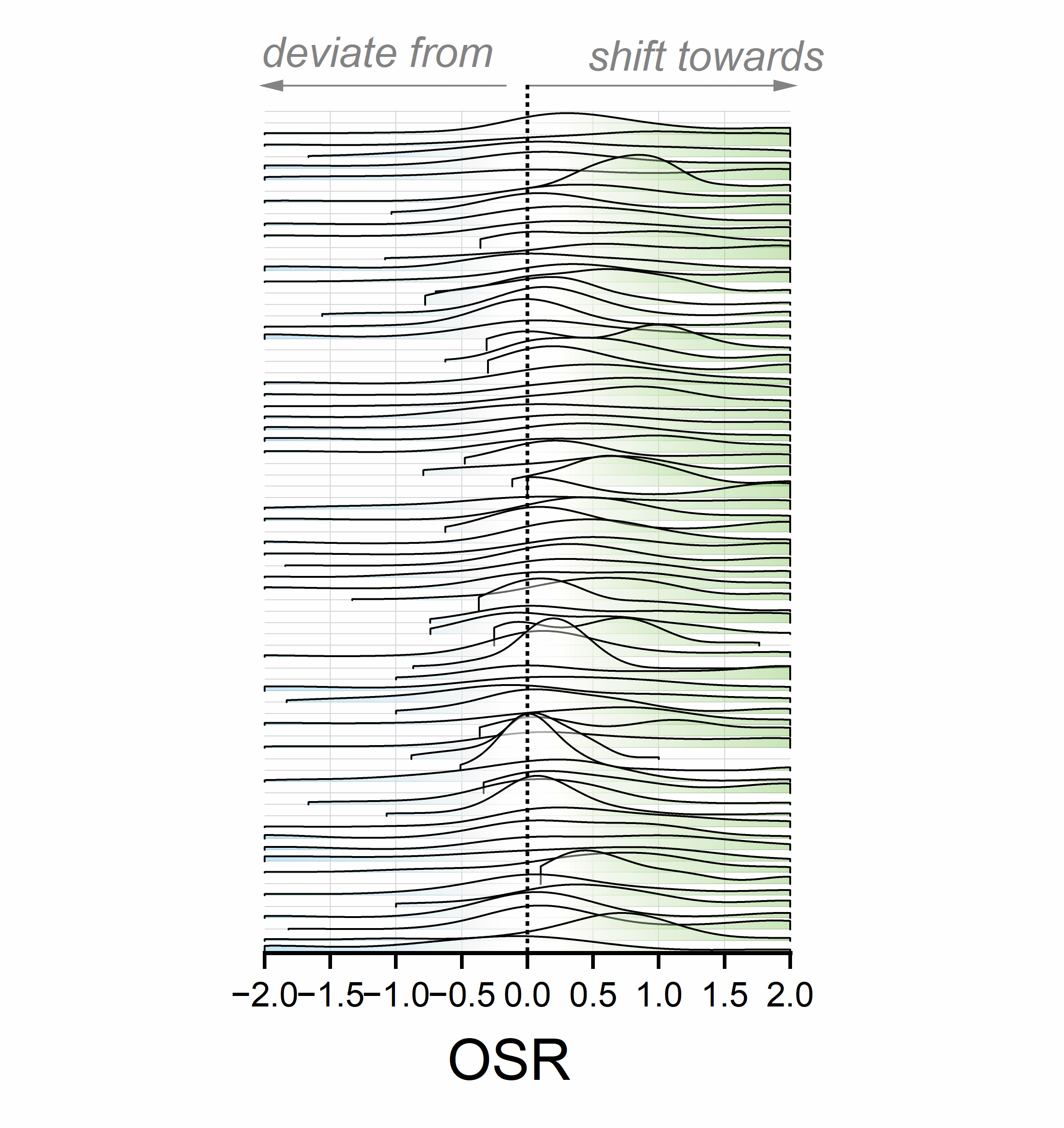

Supplement: S1 Fig — (TIF) [file pcbi.1013732.s004.tif]
