# Supplementary material for: Do we advise as one likes? The alignment bias in social advice giving
Source: PLoS Comput Biol. 2025 Dec 2;21(12):e1013732. doi: 10.1371/journal.pcbi.1013732 (PMC12688123; doi:10.1371/journal.pcbi.1013732)
Supplement: Attachment — Submitted filename: Response to Reviewers.docx [file pcbi.1013732.s006.docx]

**Dear Prof. Zhiyi Chen**

Thank you for allowing us to revise and re-submit our manuscript “Do we advise as one likes? The alignment bias in social advice giving” (**PCOMPBIOL-D-25-00735**). We want to express our sincere appreciation to the reviewers for their perceptive remarks and valuable recommendations on our manuscript. We hope the reviewers find that we have addressed each of the comments and that you agree that our revised manuscript has significantly improved. We attach the manuscript with all changes indicated in blue. Below, we provide responses to each of the reviewers’ comments.

**Reviewer #1:**

*The article offers an interesting account of four experiments seeking to understand the social mechanisms behind advice-giving behavior. The statistical analysis is well done and the series of experiments build on one another. I have three areas of concern, in the following order: First, the alignment of the research aims and the experimental design, second the interpretation of the results, and third the explanation of the series of events in the studies.*

**Response**: We thank the Reviewer for their positive feedback on our research. We have fully considered all comments and suggestions and address each of them here below.

**Comment (1)** *Alignment of aims and design: I appreciate the article weighing the distinctions among rejection, congruence, and inaccuracy as distinct factors in advice-giving. However, I am concerned that the real estate game as described does not align with a study of social advice per se, which the study describes as "informationally rich" and potentially influenced by "our need to connect with others".* *The study describes a low-information, low-sociality environment, with no social connection possible. Relationships by their nature involve multiple rounds of interaction, and advice giving in an interpersonal connection setting calls upon this relational history and mutual understanding. It is unclear how these traits of social advice and relationships map into a game wherein an individual is seeking to guess a numerical value and express confidence in their judgement**, with and without an additional piece of information. It seems more likely to test a version of economic decision-making.*

*I think I have read correctly that the tasks are performed with a few iterations at most and with no tracking in the interface showing performance and alignment that an advisor could then be understood to be able to analyze and learn from; this kind of design would address some of my concerns (e.g., it makes learning and rational adaptation more possible) but not all of my concerns given the lack of richness, lack of information / context, and lack of sociality that would persist.*

**Response**: We sincerely thank the reviewer for this thoughtful comment which raised a crucial concern on whether our experimental design effectively created a valid social context for measuring social advice. Below we respond to each point in turn:

First, definition of “social advice” vs. “non-social advice”. Our working definitions clarify the contrast between conditions: *social advice* occurs when advisors see the advisee’s expressed judgement before offering advice, while *non-social* advice occurs when advisors give advice without knowledge of the advisee’s opinion (to measure participants’ personal/independent opinions). This definition aligns directly with our core question: how does exposure to the advisee’s opinion give rise to alignment bias in advice giving? However, as the reviewer noted, our original manuscript did not sufficiently clarify that both the non-social and social advice-giving scenarios occurred within a real-time interactive context. To address potential concerns regarding the contextual settings of our studies, we first add a concise summary in the following sections.

**1. Introduction** (Lines 131-134):

“Across our studies, we simulated a real-time interaction context in which participants believed they were advising a human partner whose performance could be influenced by their advice and who would provide a subjective evaluation of advice after interaction. In Study 1 (*n* = 73) and Study 2 (*n* = 62), participants were tasked with providing advice independently and after observing the advisee’s opinions (either congruent or incongruent with participants’ initial opinions) in two sequential sessions.”

**2. Study 1** (Lines 155-157):

“In Study 1, participants were led to believe, through a deceptive instruction, that they would engage in real-time interaction with an advisee throughout both sessions of the task. Specifically, they were tasked to provide advice on investment decisions independently and after observing an advisee’s opinions, in two separate sessions.”

**6. General Discussion** (Lines 1130-1132):

“In our work, we simulated a real-time interaction context that included social consequences (advisees’ acceptance or rejection of advice) to examine whether advisors naturally tend to align their advice with advisees’ opinions when given access to them. Our findings demonstrated that, advisors have a robust propensity to align their advice with the opinions of advisees, …”

Second, in response to the concerns “*the study describes a low-information, low-sociality environment, with* *no social connection possible*” and “*relationships by their nature involve multiple rounds of interaction, and advice giving in an* *interpersonal connection setting calls upon this relational history and mutual understanding*”, we recognize that we had not effectively conveyed how we created the real-time interactive context in our studies, and whether participants indeed perceived the task context as social. We fully acknowledge that this perception was essential for drawing inferences about the social underpinnings underlying alignment bias.

In our studies, we employed background settings and cover story to induce participants’ perceptions of advisees’ presence and the real-time interaction with them, which is now described in detail and introduced in the Method sections of Study 1 and 2 as below.

**2.1.3 Experimental Task** (Lines 192-219):

“Before the experiment, two participants entered the lab at the same time and received the study instructions together. In this verbal instruction, they were informed that each participant would be assigned either the role of advisor or advisee, to convince them that they were indeed playing a real-time interaction game. The advisee’s bonus depends directly on the number of wagers won through their decisions, with the advisor’s role being to assist the advisee in maximizing winnings by providing advice. Both players completed two sessions involving identical stimuli, with the later session serving a second chance to revise decisions or advice (this was designed to conceal the true purpose of the manipulation). During the game, neither players would receive feedback on their accuracy or performance. After this verbal introduction, participants entered separate booths and received role-specific instructions. In fact, all participants were assigned to the advisor role and read the cover story as below.

‘In both sessions, the advisee will first provide his/her initial opinion in each trial and then receive your advice immediately. They can decide whether to accept it as their decision in that trial (or reject it but maintaining the initial opinion). In Session 1, you are required to provide your advice to the advisee right after the display of an estate photo and a price. In Session 2, you will be additionally presented with advisee’s opinion recorded in Session 1 in each trial. After that, you provide your advice. Before being informed of final performance in the task, the advisee would provide a subjective evaluation of your advice on a scale from 1 to 10, which was directly converted into your payment bonus (1 to 10 CNY).’

In fact, the subjective evaluations from advisees were randomly generated. Notably, we explicitly informed participants that neither the advisor nor the advisee would receive performance-related feedback. This simulated real-world advice-giving contexts in which the accuracy of advice and the consequence of its adoption are not immediately verifiable. It also mitigated the possibility that alignment merely reflected an attempt to avoid evaluative penalty for misaligned that proved to be incorrect.”

**3.1.2 Experimental Task** (Lines 508-517):

“Different from Study 1, four participants entered the lab at the same time, to make them believe that there were two advisor-advisee pairs (hence two potential advisees). This was designed to enable the manipulation regarding a “new” advisee in Session 3. Participants were jointly introduced to the study by the experimenter and read the cover story as we used in Study 1, including the information that they would receive the advisee’s subjective evaluation at the end of the task. However, they were informed that their bonus would be calculated based on the trial-by-trial performance of advice (i.e., accuracy × wager, with accuracy coded as –1 for incorrect and 1 for correct). The overall performance score will be linearly rescaled to a 0–10 range and converted to payment bonus (1 to 10 CNY), which was added to the base payment.”

Notably, participants’ perceptions of advisee’s presence and the direct interaction with this advisee were validated by their responses in the post-task debriefings. No participants across Studies 2-4 expressed skepticism regarding the authenticity of the advisees when they were asked “Did you have any thoughts or impressions about the advisee player you were interacting with during the task?”) in the post-task debriefings, as described below.

**3.1.3 Post-task survey** (Lines 544-546):

“… in response to experimenter’s open-end asking, ‘Did you have any thoughts or impressions about the advisee player you were interacting with during the task?’”

**3.1.1 Participants** (Lines 504-505), **4.1.1 Participants** (Lines 659-661), and **5.1.1 Participants** (Lines 1027-1029):

“… No participants expressed suspicion regarding the authenticity of the advisee in the post-task survey, and thus no exclusion was made on this basis.”

Third, in response to the reviewer’s comment that “*It is unclear how these traits of social advice and relationships map into a game wherein an individual is seeking to guess a numerical value and express confidence in their judgement, with and without an additional piece of information*. *It seems more likely to test a version of economic decision-making*”, we would like to first clarify our rationale for employing an economic decision-making task. Then, we explain why the present study has social features and does not reduce to a “standard” economic decision-making task.

We used standardized economic decision-making task, involving binary judgements and confidence estimations, to serve as the task context. This allowed for a rigorous manipulation of the initial congruency between participants’ personal opinions and those of the advisees, which was a key factor in our design. This manipulation enabled us to examine whether advisors tend to re-align with advisees’ opinions when they are initially incongruent.

The “social” features of advice giving in our studies were established by role framing and the real-time interaction context. First, participants were led to believe they were situated in a real-time connection with a human partner through the instructions. They were explicitly assigned the role of advisor (with the partner as the advisee) and informed that their goal was to help the advisee perform better, rather than making economic decisions for themselves. Second, the social connection was further reinforced by outcome interdependence grounded in the role framing. Advisors believed that their advice would influence the advisee’s performance (if accepted), and the advisee would in turn provide a subjective evaluation of the advice at the end of the task. This evaluation either determined the advisor’s monetary reward (Study 1) or purely served as social approval (Studies 2–4). Consequently, participants were motivated to consider not only how their advice would affect the advisee’s performance, but also the extent to which the advisee appreciate their advice. In this sense, the advisee’s opinions (displayed in Session 2) could also serve as social cues, enabling advisors to predict advisees’ attitudes towards their advice by comparing it with these opinions, rather than mere numeric cues.

**In summary, our studies established a situated social connection between participants and an (alleged) advisee, perceived to be a real social partner, based on real-time interaction context and role framing. These manipulations prompted participants to engage in interpersonal considerations, especially when they were shown the advisee’s initial opinions.** **However, standard economic decisions, involving rational and informational processing, typically exclude such social cognitive processes. This distinction could also address the reviewer’s major concern regarding the perceived “*low sociality*” and “*no social connection*” in our studies.** We have incorporated a direct clarification in the Methods section explaining that our study could not reduce social advice-giving behavior to standard economic decision-making as below.

**2.1.3 Experimental Task** (Lines 220-224):

“Therefore, the perception of real-time interaction with a human partner, combined with role framing that introduced reward interdependence between advisor and advisee, helped construct a situated social connection in our experiments. These manipulations ensured that the advice-giving behavior observed in our studies reflected a social process, rather than a purely economic decision as in non-social situations.”

Finally, in response to the reviewer’s comments that “*the task may involve only a few iterations, without any interface for tracking performance or alignment feedback that advisors could learn from, ; this kind of design would address some of my concerns (e.g., it makes learning and rational adaptation more possible)*”, we would like to clarify that the goal of Studies 1 and 2 was to examine the general tendency of advisors to align with advisees’ opinions (i.e., alignment bias). To observe this tendency in its natural and unbiased form, neither performance nor alignment feedback was provided, as any learning signal might interfere with the spontaneous alignment behavior we aimed to capture.

To address this concern, we have incorporated the explanations in the following section of Study 1 as below.

**2. Study 1** (Lines 161-163):

“Notably, we did not incorporate any feedback from advisees in order to capture alignment bias in its natural form that was not induced by additional information.”

**3.1.2 Experimental Task** (Lines 214-219):

“Notably, we explicitly informed participants that neither the advisor nor the advisee would receive performance-related feedback. This simulated real-world advice-giving contexts in which the accuracy of advice and the consequence of its adoption are not immediately verifiable. It also mitigated the possibility that alignment merely reflected an attempt to avoid evaluative penalty for misaligned that proved to be incorrect.”

Building on the observed presence of alignment bias, we introduced trial-by-trial feedback in Studies 3 and 4 to further investigate the potential mechanism of its emergence. In these studies, participants received explicit feedback on each trial (acceptance vs. rejection), with advice being either aligned or misaligned with the advisee’s opinion. This feedback structure enabled participants to adjust their advice-giving tendencies in an acceptance-maximizing (rejection-minimizing) manner. As reported in “**4.2 results**” and “**5.2 results**”, participants adjusted their behavior in response to advisees’ varying preferences, consistent with the idea that they *spontaneously* adapted to this social signal.

Importantly, we did not provide aggregated or summary feedback linking alignment to acceptance (e.g., “Your advice is aligned with the advisee’s opinion. Your advice is accepted”), as doing so might have artificially directed participants’ attention to this contingency. Instead, our goal was to examine whether participants would spontaneously adjust their behavior to navigate acceptance. To clarify this rationale and avoid potential misunderstanding, we have now added further explanation of the feedback structure in the revised manuscript.

**4.1.2 Experimental Task** (Lines 680-683):

“We presented feedback without displaying the advice type in that trial, to capture value-based learning in its natural form, allowing advisors to spontaneously notice and adapt to the contingencies between advice type and feedback.”

**Comment (2)** *Interpretation of results: In study 1, the article states: "Participants were informed that their payments were partially determined by the advisees given their subjective evaluations on the advice at the end of the task." It seems reasonable for participants to expect to be penalized by the subjective evaluation for misalignment if they are not correct, and rewarded for alignment regardless of whether they were correct or not, making misalignment a higher-risk state. I believe M7 seeks to evaluate this interpretation, however this distinction and the problem it poses for study 1 is not addressed.*

**Response**: We thank the reviewer for raising this important concern. The reviewer proposed a plausible alternative explanation for the pattern of unselective re-alignment to the advisee’s opinion—namely, that it might reflect participants’ concern about the evaluation risk associated with influencing advisees to make incorrect decisions.

We wish to clarify that our designs in Studies 1 and 2 could effectively preclude this possibility. Specifically, before the task began, participants were informed that: neither player would be shown immediate feedback on the decisions, and advisees’ evaluation of advice was required to be provided before they were informed of their final performance. This eliminated the possibility that advisee would evaluatively penalize participants for the advice inaccuracy. This information is now explicitly noted in the Method section of Study 1 as below.

**2.1.3 Experimental Task** (Lines 200-201):

“During the game, neither players would receive feedback on their accuracy or performance.”

To further prevent potential misinterpretation that participants’ bonuses in Study 1 were contingent on their actual performance, we added the following clarification to the cover story, as detailed below.

**2.1.3 Experimental Task** (Lines 214-219):

“Notably, we explicitly informed participants that neither the advisor nor the advisee would receive performance-related feedback. This simulated real-world advice-giving contexts in which the accuracy of advice and the consequence of its adoption are not immediately verifiable. It also mitigated the possibility that alignment merely reflected an attempt to avoid evaluative penalty for misaligned that proved to be incorrect.”

**Comment (3)** *Interpretation of results: The article describes its aim as "to investigate whether advisors gradually aligned their advised investments to align with advisees’ risk preference". However, the description of the stimuli does not incorporate discussion of risk preference, only the dollar figure guess. In addition, it is unclear how the study tests notions of gradual alignment in a two-round interaction.*

**Response**: We thank the reviewer for raising this crucial question. We would like to clarify that the investigation of this gradual alignment was not conducted at the within-trial level (as stated by the reviewer by “*a two-round interaction*”), but at the between-session level.

Specifically, with participants’ level of investment magnitude in Session 1 serving as baseline, we examined whether advisors adjusted this level to that of advisee in Session 2 (where advisee’s risk preference was shown by their overall wagering magnitude). The details of how we manipulated advisees’ risk preference (by requiring the advisee to provide their level of investment, i.e., post-decision wagering), and how we examined its influence on advice-giving behaviors have now been revised to make it more explicit.

**2.1.3 Experimental Task** (Lines 229-236; Lines 248-253):

“In the social scenario (Session 2), participants observed all the stimuli again and provided advice after displayed with the interacting advisee’s initial opinions (both judgements and investments) on the stimuli, except for 20% of trials (14 trials) where advisees’ opinions were masked (identical to the *non-social* scenario). The inclusion of masked trials within the social advice-giving scenario was designed to investigate how advisees’ behavioral preferences (specifically, risk preference) shaped advice giving while isolating the trial-by-trial influence of opinions from advisees (see **2.1.6** for details).”

“Advisees’ investments were all artificially generated from normal distributions, *Normal*(10,10) or *Normal*(50,10), distinguish their risk-avoiding or risk-seeking risk preferences as a between-subject variable. These distributions were designed to contrast with participants’ typical investments (around 30) in the non-social session, thereby allowing for adaptations in the social session. In other words, this setup aimed to investigate whether advisors gradually aligned their advised investments to align with advisees’ risk preferences on the basis of observations of advisees’ decision.”

**2.1.6 Design, measurements, and analytical plans** (Lines 321-328):

“In addition to investigating the presence of alignment bias towards advisees’ opinions, we further explored if advisors implicitly internalized and aligned towards the behavioral preferences of their advisees, revealing a subtle aspect of alignment bias. Specifically, we compared advisors’ investment magnitude between Session 2 and Session 1 specifically in trials where advisees’ opinions were masked (to isolate the influence of trial-by-trial opinions from the advisee) and examined whether the direction of this effect varied depending on the conditions of advisees’ risk preferences (risk-avoiding vs. risk-seeking).”

**Comment (4)** *Interpretation of results: Study 2 reiterates the interpretation from Study 1 that tendency to align with incongruent opinions as "alignment bias" and proposes to distinguish this from two other potential interpretations -- opinion contagion and anchoring. This makes sense, however the design seems insufficient to eliminate contagion and anchoring as interpretations --- or as more simple information-seeking / rational choice behaviors as mechanisms given the low information conditions and few iterations.*

**Response**: We thank the reviewer for raising this important concern. Though probable, these interpretations rest on a common assumption: alignment bias simply reflects strategies for calibrating advice or being unconsciously influenced by advisees’ opinions, while did not involve any social factors. However, we believe that the findings from Study 2 offer compelling evidence against these alternative accounts.

In Study 2, we implemented a subtle yet important manipulation in a third-session (Session 3) following Session 2. In Session 3, advisees’ opinions were no longer presented, replicating the non-social condition of Session 1. Crucially, a between-subjects manipulation was introduced in Session 3: half of the participants were informed they would continue advising the same advisee from Session 2, while the other half were told they would be advising a new advisee.

This design enabled us to test whether alignment bias depends on social factors tied to the ongoing social connection. We hypothesized that if alignment bias reflected a social process, participants in the new-advisee condition would show weaker maintenance of re-alignment than those in the same-advisee condition, as no social motive persisted in this session to sustain prior re-alignment. In contrast, if alignment bias were solely driven by the low-level or unconscious processes, as suggested by the reviewer, no difference in the persistence of re-alignment would be expected between the new- and same- advisee conditions.

Indeed, we found that participants in the new-advisee condition exhibited a significantly greater decrease in judgement congruency (Session 3 – Session 2) compared to those in the same-advisee condition. Additionally, the re-aligned judgements observed in Session 2, maintained to a smaller degree in Session 3 in the new-advisee condition compared to the same-advisee condition. Importantly, our data also demonstrated that this observation could not simply be attributed to a greater motivation to maintain behavioral consistency when interacting with the same advisee.

To explicitly clarify how we ruled out these alternative interpretations and to enhance the logical progression from our rationale to the expected results, we have reorganized the following sections accordingly.

**1. Introduction** (Lines 139-143):

“In Study 2, we investigated the generalizability of this bias under a performance-based incentive structure and examined whether advisors’ re-alignment to advisees’ opinions reflected a social basis (i.e., normative conformity), or merely reflected non-social processes and unconscious influences (e.g., opinion contagion [27] or anchoring effects [28]).”

**3. Study 2** (Lines 473-476; Lines 480-488):

“However, it remained unclear whether this conformity reflected a social basis or instead arose from non-social processes (e.g., information seeking, copying strategies) and unconscious influences (e.g., opinion contagion [27] or anchoring effects [28]).”

“To disentangle alignment bias from non-social processes and unconscious influences, we introduced an additional session (Session 3) in Study 2, where participants gave advice independently again, while being informed that their advice would be provided either to the *same* advisee from Session 2 or a *new* advisee. Participants advising a new advisee were expected to exhibit a greater decline in re-alignment with the advisee’s opinions from Session 2 compared to the participants in the same-advisee condition. Conversely, if alignment bias was solely due to non-social processes or unconscious influences, no difference in the decline of re-alignment between these conditions would be anticipated.”

Additionally, we further incorporate supplemental analyses aimed at strengthening our conclusion on the social basis of the conformity to advisees’ opinions, and re-organized our results as below.

**3.1.4 Design, measurements, and analytical plans** (Lines 555-568):

“Data from Session 3 were analyzed to investigate whether the conformity to advisees’ opinions involved social motivations (i.e., normative conformity) or instead reflected non-social processes and unconscious influence. Specifically, we employed a linear mixed-effects model to test whether the decrease in judgement congruency with advisees’ Session 2 opinions, from Session 2 to Session 3, differed between participants advising the same advisee versus a new advisee. To further assess whether this difference reflected a decreased tendency to maintain re-alignment, we conducted a follow-up analysis focusing on trials where participants had previously re-aligned with the advisees’ judgements in Session 2, comparing the advisee conditions.

Additionally, the observed difference in re-alignment maintenance might alternatively reflect a greater motivation to maintain behavioral consistency when interacting with the same advisee. To examine this possibility, we further constructed a linear mixed-effects model comparing the likelihood of repeating the same judgement from Session 2 across advisee conditions.”

**3.2.2 The conformity to advisees’ opinions reflects a social basis** (Lines 611-622):

“Next, we examined the normative features of the conformity to advisees’ opinions by analyzing advice-giving patterns in Session 3. Consistent with our hypothesis, from Session 2 to Session 3, participants in the new-advisee condition exhibited a greater decrease in judgement congruency compared to those in the same-advisee condition (*β* = 0.26, *z* = 2.53, 95% CI = [0.06, 0.46], *p* = 0.01). Moreover, the re-align judgements observed in Session 2 were less likely to be maintained by participants in Session 3, in the new-advisee condition than by those in the same-advisee condition (*β* = -0.49, *z* = -2.48, 95% CI = [-0.87, -0.10], *p* = 0.01). Furthermore, this between-condition difference could not be attributed to a motivation to preserve a self-consistent image by maintaining previous judgements, as participants in the same-advisee condition did not show greater cross-session consistency in their advised judgements compared to those in the new-advisee condition (*β* = 0.20, *z* = 1.40, 95% CI = [–0.08, 0.48], *p* = 0.16).”

**3.3 Discussion** (Lines 628-633):

“Importantly, the maintenance of re-alignment with advisees’ opinions diminished when participants switched to provide advice to a new advisee, compared to the advisee they had previously interacted with. This finding ruled out the possibility that advisors’ re-alignment to advisees’ opinions merely resulted from information-seeking behaviors or having influenced by advisees’ opinions unconsciously, instead emphasizing its normative nature.”

**Comment (5)** *Interpretation of results:* *The success of M7 and general persistence of desire for advice acceptance is interesting, but ultimately it is situated inside a series of studies that are structured to evaluate economic decision making rather than social relationships.*

**Response**: We thank for the reviewer’s appreciation of the success of our model M7. Beyond those has been clarified regarding the socialness of our studies as in Response #1 and #4, we agree with the reviewer that real-life multi-round social interactions can be tested in the future and see if our model-based results would still hold. We have now added this aspect to the Discussion in the paragraph where we address the limitations.

**6. Discussion** (Lines 1252-1261):

“Finally, in the current study, we induced participants’ perception of engaging in social interactions with a human partner through physical contact and task-based reward interdependence, to ensure that participants believed they were receiving advice feedback in real social encounters. Although this approach proved effective, the extent to which our findings on alignment bias generalize in real-world social relationships (e.g., friendships), where advice feedback may carry greater value for individuals, remained to be investigated. Addressing this issue is essential for clarifying the social complexity of alignment bias, and future research should examine the interplay between the prosocial motivation to benefit close others and having favorable social feedback during social interaction.”

**Comment (6)** *Clarity of design overall:* *The essence of my concern here is that it is not clear what participants know and when they know it. For example, for study 1 the article describes realignment as occurring "even when incorrect" and evaluating whether advisor realignment was "contingent on the accuracy of those opinions" -- however, it is not clear how and when the participants knew the actual value, or how the magnitude of the incentive for making a good guess compared to the incentive of the advisee giving them a positive rating.*

*The same issue persists for study 2, which describes how "the inclination of re-align persisted both when advisee’s judgement was correct...and when it was incorrect." Did advisors re-align to match the opinions of advisees despite knowing that the advisee was incorrect and that the advisee would have no influence on their reward? The incentive structure in the design of study 2 further diverges from the incentive structure of more naturalistic advice-giving scenarios, which socially reward advice acceptance and accuracy in varying degrees by context with the advisee as a substantial factor. In general, the reward conditions and subjects' understanding of the reward conditions needs to be made very clear for each study.*

**Response**: We appreciate the reviewer’s thoughtful comments and fully acknowledge the need to clarify the reward conditions and subjects’ understanding of the reward conditions for each study. We address the reviewer’s concerns in three parts.

First, in response to the comment “*it is not clear how and when the participants knew the actual value*”, participants were informed that both advisor and advisee would not receive immediate feedback on their decisions and advice throughout the task. This information is now incorporated in the description of the cover story as below.

**2.1.3 Experimental Task** (Lines 200-201):

“During the game, neither players would receive feedback on their accuracy or performance.”

Therefore, any analysis of whether re-alignment occurred “even when incorrect” refers to the actual accuracy (via the ground-truth knowledge for us as experimenters) of the advisee’s judgement, as coded based on whether the actual price was higher or lower than the stimulus price. To facilitate interpretability, we have now corrected the expression we used as below.

**2.1.6 Design, measurements, and analytical plans** (Lines 361-363):

“Accuracy was coded according to the ground truth—specifically, whether the stimulus price was higher or lower than the actual price.”

**2.2.2 Advisors exhibited an unselective inclination to re-align with advisees’ opinions** (Lines 411-412):

“Next, we investigated whether the tendency of advisors to re-align with advisees’ judgements depended on their actual accuracy”

Second, in response to the comment “*how the magnitude of the incentive for making a good guess compared to the incentive of the advisee giving them a positive rating*” and “*In general, the reward conditions and subjects' understanding of the reward conditions needs to be made very clear for each study*”, we understand the reviewer’s concern about participants’ potential trade-off between providing accurate advice and securing a favorable evaluation from their advisees (which was directly linked to participants’ final payment) by avoiding misalignment.

To preclude this possibility and further examine the generalizability of alignment bias and its intrinsic motivational basis, we used a performance-based incentive structure in Study 2 (as well as Studies 3-4) aiming to replicate the findings we observed in Study 1. This incentive structure exclusively incentivized advice accuracy. In other words, in Studies 2–4, participants had no external monetary incentive to secure favorable subjective evaluations from advisees. Thus, in Study 2, the observed unselective re-alignment or acceptance-maximizing behavior were unlikely to reflect strategic economic considerations but instead suggested the operation of intrinsic social motivations.

We have now clarified the incentive structures in Studies 1 and 2 and how these were communicated to participants through the cover story in the Method sections. Additionally, we have elaborated on how the reward structure employed in Studies 2–4 enabled us to isolate and detect the intrinsic motivational basis of advice-giving behavior.

**2.1.3 Experimental Task** (Study 1, Lines 192-219):

“Before the experiment, two participants entered the lab at the same time and received the study instructions together. In this verbal instruction, they were informed that each participant would be assigned either the role of advisor or advisee, to convince them that they were indeed playing a real-time interaction game. The advisee’s bonus depends directly on the number of wagers won through their decisions, with the advisor’s role being to assist the advisee in maximizing winnings by providing advice. Both players completed two sessions involving identical stimuli, with the later session serving a second chance to revise decisions or advice (this was designed to conceal the true purpose of the manipulation). **During the game, neither players would receive feedback on their accuracy or performance.** After this verbal introduction, participants entered separate booths and received role-specific instructions. In fact, all participants were assigned to the advisor role and read the cover story as below.

‘In both sessions, the advisee will first provide his/her initial opinion in each trial and then receive your advice immediately. They can decide whether to accept it as their decision in that trial (or reject it but maintaining the initial opinion). In Session 1, you are required to provide your advice to the advisee right after the display of an estate photo and a price. In Session 2, you will be additionally presented with advisee’s opinion recorded in Session 1 in each trial. After that, you provide your advice. **Before being informed of final performance in the task, the advisee would provide a subjective evaluation of your advice on a scale from 1 to 10, which was directly converted into your payment bonus (1 to 10 CNY).**’

In fact, the subjective evaluations from advisees were randomly generated. **Notably, we explicitly informed participants that neither the advisor nor the advisee would receive performance-related feedback.** This simulated real-world advice-giving contexts in which the accuracy of advice and the consequence of its adoption are not immediately verifiable. **It also mitigated the possibility that alignment merely reflected an attempt to avoid evaluative penalty for misaligned that proved to be incorrect.**”

**3. Study 2** (Lines 489-495):

“Additionally, to address the possibility that re-aligning with advisees’ opinions unselectively served merely as a strategy to conserve effort on advice calibration, as well as to further examine the generalizability of alignment bias and its intrinsic motivational basis, we replaced the evaluation-based incentive structure used in Study 1 with a performance-based structure in Study 2. In this design, participants’ bonuses were directly linked to the quality of their advice, thereby eliminating any economic motivations to pursue unselective re-alignment.”

**3.1.2 Experimental Task** (Study 2, Lines 511-517):

“Participants were jointly introduced to the study by the experimenter and read the cover story as we used in Study 1, including the information that they would receive the advisee’s subjective evaluation at the end of the task. However, they were informed that their bonus would be calculated based on the trial-by-trial performance of advice (i.e., accuracy × wager, with accuracy coded as –1 for incorrect and 1 for correct). **The overall performance score will be linearly rescaled to a 0–10 range and converted to payment bonus (1 to 10 CNY), which was added to the base payment.**”

**Comment (7)** *Clarity of design overall: I further noted the usage of random variation from the actual pricing of real estate as part of the experimental manipulation; did the algorithmically-generated figures conform to common expectations around what a price looks like (e.g. round numbers, common digits), or did the numbers potentially appear artificial? I also observe that the study filtered out people who detected the manipulation: were people who answered this question informed that their reward would not be influenced by the answer to the question ? What was the magnitude of individuals who were excluded due to this? Were there systematic differences in the performance of these more insightful individuals with respect to the task?*

**Response**: We appreciate the reviewer’s valuable suggestion regarding the need to clarify the description of the stimuli. All stimulus prices in the risk investment game were generated by adding or subtracting 20% from the actual price of estate properties, and the final values were rounded to the nearest whole number in our procedures. We have now added a description of this setting to the Method section of Study 1 as below.

**2.1.4 Stimuli** (Lines 259-261):

“The stimulus price was either 20% higher or lower than the real price of a property, and all values were rounded to the nearest whole number to ensure consistency with common pricing conventions.”

Regarding the concern about participants’ detection on deceptive manipulations of the alleged advisee, we have addressed this in our post-task survey. Specifically, after participants received their payments, they were asked the following open-ended question: “*Did you have any thoughts or impressions about the person you were advising during the task?*”. Participants were not informed in advance that they would be asked this question, and we made no indication that their responses would have any influence. This approach was intended to minimize response bias and encourage honest answers. We incorporate the details of this post-task survey section in Study 2 as below.

**3.1.3 Post-task survey** (Lines 544-550):

“…, in response to experimenter’s open-end asking, ‘Did you have any thoughts or impressions about the advisee player you were interacting with during the task?’. Notably, Participants were not informed in advance that they would be asked this question, and we made no indication that their responses would have any influence, in order to minimize response bias and encourage honest answers. Those who indicated that they perceived the advisees as fictitious would also be excluded.”

Notably, in Studies 2–4 (where post-task surveys were implemented), no participants expressed doubts about the authenticity of the advisee (e.g., question whether the advisee was simulated) and thereby excluded. We have directly declared this as below.

**3.1.1 Participants** (Lines 504-505), **4.1.1 Participants** (Lines 659-661), and **5.1.1 Participants** (Lines 1027-1029):

“… No participants expressed suspicion regarding the authenticity of the advisee in the post-task survey, and thus no exclusion was made on this basis.”

**Comment (8)** *Document preparation notes:*

*Line 119 -- "whether acceptance-seeking intentions underlying this bias."*

*Line 159 -- "a sample of at least 59 participants were required"*

*Line 500 -- "the inclination of re-align persisted"*

**Response**: We have corrected these errors as below:

Line 119*-- "whether acceptance-seeking intentions underlying this bias."* was removed.

Line 176: Revised to “a sample of at least 59 participants was required.”

Line 584: Revised to “the inclination of re-alignment persisted.”

**Reviewer #2:**

*I thank the authors for producing this manuscript, which constitutes a timely addition to an important and gravely understudied topic. I also thank the editor for the opportunity to review this work. I want to formally state as well that I have not used AI for any part of this review process. Here is a more in-detail account of my views on each part of the manuscript.*

**Response**: We thank the Reviewer for their positive feedback on our research. We have fully considered all comments and suggestions and address each of them here below.

**Comment (1)** *GRAPHICAL ABSTRACT: I believe it is very good, but it may be a bit opaque for people who are not initiated in the topic. The upper part (studies 3 and 4) is super clear, but I think the lower part (studies 1 and 2) is a bit less transparent for the untrained eye. I do not request any modifications because if I'm the public for this abstract, then it's spot on. But in case you may want a more accessible item, maybe consider rethinking the lower part.*

**Response**: We have revised the lower part (corresponding to Studies 1 and 2) and added a summarized note of this graphical abstract to improve clarity and accessibility for readers who may be less familiar with the topic.

*Note.* This illustration summarizes the key findings of our study. In Studies 1–2, we identified an alignment bias in social advice giving, whereby advisors adjusted their advice toward advisees’ initial opinions. We further demonstrated that this bias was driven by normative conformity, as it disappeared once the interacting advisee changed. In Studies 3–4, we discovered advisors’ behavioral adaptations that maximized advisees’ acceptance and minimized rejection of advice, providing a value-based learning perspective on the emergence of alignment bias.

**Comment (2)** *INTRODUCTION: The introduction is principled and complete. The question of the relevance of advice-giving and the biases that intervene when crafting advice are well defined. The one big omission that I think should be addressed for clarity and completion is a mention to *how* advice-givers consider their *own* knowledge when deciding whether or not to give advice. This was studied extensively by Anllo, Hertz and cols (see https://www.nature.com/articles/s44271-024-00175-5). Incorporating this angle into the introduction or discussion is I believe of crucial importance, because that work outlies an adversarial setup in which participants have to weight their own knowledge about value versus different social cues at the time of giving advice (an issue that is complementary to the researchers' question to a very high degree, because it touches directly upon the key question of whether advice is useful or not). Another important point is that the idea that advice-gives could be influenced by the very people they are trying to influence (the advisees) is important and needs to be studied, but it is not new as it has been considered (although not directly) in the work by Hertz et al 2020, where advice-givers seek to sway advisees (although my remark here is less severe because the authors do come back to this paper during their Discussion).*

**Response**: We thank the reviewer for providing these valuable insights. We fully agree that incorporating the perspective of how advisors weigh their own knowledge against social cues during advice-giving is critical to providing a comprehensive theoretical background. The work by Anllo, Hertz, and colleagues (2024) highlights how consideration of social consequences can shape the advice-giving behaviors. To address this, we have incorporated this valuable evidence into the introduction section as below.

**1. Introduction** (Lines 116-122):

“Recent research directly shows that individuals’ willingness to provide advice reflects a complex interplay between informational and social factors [25]. People become more inclined to share their opinions as advice as their knowledge increases, and this tendency is further amplified when social rewards are introduced, namely, when they are informed that their advice and performance ratings will be visible to future advisees. This finding suggests that advice-giving, often seen as an information-driven behavior, can be shaped by anticipated feedback from advisees.”

We also appreciate this reviewer’s comment regarding the novelty and theoretical contribution of our central claim—that advisors may be influenced by the very individuals they seek to influence. Indeed, prior work has touched on this idea. To better position our contribution in relation to prior work, we have revised the relevant descriptions as below.

**1. Introduction** (Lines 123-126):

“This further raises a striking yet plausible possibility that has been suggested by previous work [4,26]: advisors—typically seen as impartial and in control of influencing others—may also be swayed by social interests and, in turn, influenced by advisees, who are conventionally seen as the ones being influenced.”

**Comment (3)** *STUDY 1-METHODS: I appreciate the a-priori power analysis, but would like to see some justification as to why a d = 0.4 was chosen, and why a power of 85% was the target.*

**Response:** We thank the reviewer for providing this valuable suggestion. We have added justifications to the manuscript in this section of Study 1 as below.

**2.1.2 Participants** (Lines 177-180):

“We selected an effect size of *d* = 0.4, following Cohen’s (1988) convention for a medium effect [29], which we considered a reasonable benchmark given the limited prior evidence. To ensure adequate sensitivity while keeping the sample size feasible, we set the power level at 85% (above the conventional 80% threshold).”

**Comment (4)** *STUDY 1-METHODS:* *I would appreciate a bit more clarity in the presentation of the design. If I understand correctly, even in the "social" condition participants are never* *in actual contact with the advisees, right? Further, you mention that you generate advisees judgements, but you also mentioned that you needed to keep the order of blocks as "isolation" then "social" because you needed the decisions and judgements of the advisees. So please exactly explain how much of the advisees judgements is being engineered or "generated". Is it just the order? Then the investments were also randomly selected from normal distributions... please attempt to explain exactly how much is "human made" and how much is "generated".*

**Response:** We thank the reviewer for raising this important question. In each testing session, two actual participants joined the study and met with each other, and they were led to believe that one of them would act as the advisor and the other as advisee. Hence, they had been in physical contact upon arrival and the instruction session.

In reality, though, both of them acted as the advisor. This procedure was designed to convince participants that the opinions from the advisee they would interact with were genuine, thereby simulating a realistic social interaction. This manipulation was implemented through a specific background setting and a cover story, which is now detailed in the Method section of Study 1 as below.

**2.1.3 Experimental Task** (Lines 192-213):

“Before the experiment, two participants entered the lab at the same time and received the study instructions together. In this verbal instruction, they were informed that each participant would be assigned either the role of advisor or advisee, to convince them that they were indeed playing a real-time interaction game. The advisee’s bonus depends directly on the number of wagers won through their decisions, with the advisor’s role being to assist the advisee in maximizing winnings by providing advice. Both players completed two sessions involving identical stimuli, with the later session serving a second chance to revise decisions or advice (this was designed to conceal the true purpose of the manipulation). During the game, neither players would receive feedback on their accuracy or performance. After this verbal introduction, participants entered separate booths and received role-specific instructions. In fact, all participants were assigned to the advisor role and read the cover story as below.

‘In both sessions, the advisee will first provide his/her initial opinion in each trail and then receive your advice immediately. They can decide whether to accept it as their decision in that trial (or reject it but maintaining the initial opinion). In Session 1, you are required to provide your advice to the advisee right after the display of an estate photo and a price. In Session 2, you will be additionally presented with advisee’s opinion recorded in Session 1 in each trial. After that, you provide your advice. Before being informed of final performance in the task, the advisee would provide a subjective evaluation of your advice on a scale from 1 to 10, which was directly converted into your payment bonus (1 to 10 CNY).’”

**However, advisees and their opinions displayed in Session 2 were in fact all programmed.** Advisees’ judgements were computer-generated, based on each participant’s personal/independent judgements (measured as their *non-social* advice in Session 1) regarding the same stimulus. This was designed to implement a key manipulation: in trials where advisee’s opinion was displayed, 50% of the advisees’ judgements were congruent with the participant’s independent judgement, and 50% to be incongruent. To enable this manipulation, participants must complete the non-social session first.

Additionally, all advisees’ investment magnitude were sampled from normal distributions—specifically, *Normal*(10,10) for risk-avoiding advisees and *Normal*(50,10) for risk-seeking advisees—so that the observed investment behaviors reflected distinct risk preferences while maintaining trial-level variability. To enhance transparency, we have clarified the generation procedure of advisees’ opinions in Study 1’s Experimental Task section as below.

**2.1.3 Experimental Task** (Lines 242-249):

“Unbeknownst to participants, advisees they interacted with were alleged. In fact, advisees’ opinions were preprogrammed (therefore deceptive approaches had been used in the experiments). Advisees’ judgements were generated according to participant’s non-social judgements in Session 1 (hence, the non-social session had to be completed before the social session), ensuring that in half of the trials wherein advisees’ opinions were presented, advisees held incongruent judgements with the advice given by participants in Session 1 (the other half of the trials were hence congruent). Advisees’ investments were all artificially generated from normal distributions, ...”

**Comment (5)** *STUDY 1-METHODS:* *When it comes to your ANOVA approach, to my knowledge the term "mixed-effects one-way ANOVA" does not exist. It appears you are conflating linear mixed-effect regressions and x-ways ANOVAS. Please clarify. You discuss several "mixed-effect" approaches, which implies you actually built a statistical model (an assumption you confirm two paragraphs later... it may be better to explain everything together in one go). However, no information is provided however as to what these models are (just presenting the models and the approach for parameter optimization would suffice).*

**Response:** We apologize for this confusion. We have now replaced all related terminology into “linear mixed-effect model”. We have incorporated the model specifications into the Supplementary Materials (S1 Table). Besides, we have incorporated the approach for parameter optimization into the “**2.1.6 Design, measurements, and analytical plans**” section. Additionally, to improve readability, we have now merged the original “**2.1.7 Data exclusions and statistical analyses**” section into “**2.1.6 Design, measurements, and analytical plans**”.

Notably, after careful consideration, we chose to include only participant and stimulus (i.e., estate photo) as random intercepts to account for the non-independence of repeated measures. This was made to ensure analytical consistency across all analyses and to improve model convergence. More complex models—including those with random slopes—failed to converge at times, resulting in inconsistent comparisons and reduced interpretability. After re-running all analyses using random intercept–only models, we reproduced results that were identical in both direction and statistical significance to those reported in the original manuscript. The revised versions of the aforementioned sections are provided below.

**2.1.6 Design, measurements, and analytical plans** (Lines 285-305):

“…Before conducting data analysis, we excluded trials where the value of the examined variable was missing, or where the value exceeded the constraint scales (e.g., investments magnitude outside the range of 1-60). Under the consideration of the fractional property, we incorporated additional exclusion criteria for OSR (see details in the next paragraph). After data exclusions, we constructed linear mixed-effect models to conduct statistical analysis using the lme4 package [30] in R (Version 4.3.1).

Generalized linear mixed-effect models were employed when the dependent variable was binary (e.g., judgement congruency or judgement re-alignment) using the *glmer*() function, assuming a binomial distribution for the response and reporting fixed-effect statistics as *z*-values. Linear mixed-effect models were used when the dependent variable was continuous (e.g., opinion congruency or OSR) using the *lmer*() function, with fixed-effect statistics reported as *t*-values along with the corresponding degrees of freedom. The lmerTest package was used to perform significance tests on the parameters yielded by the model [31]. Post-hoc comparisons were performed using the emmeans package [32]. Participants and stimuli (i.e., estate photos) were modeled as random intercepts across all analyses. All variables were mean-centered. We used the default ‘nloptwrap’ parameter optimization method to fit the models. The specifications of all mixed effect models used in the present study are provided in S1Table. For statistical analysis involving only one observation per participant (e.g., individual-level computational parameters), we employed conventional statistical approaches using Jamovi computer software [33].”

**Supplementary Materials (S1Table.** **Mixed-effect models specifications.)**:

2.2.1 Advisors aligned their advice towards advisees’ opinions

Judgement congruency ~ Session (1 vs. 2)

Opinion discrepancy ~ Session (1 vs. 2)

Investment magnitude ~ Session (1 vs. 2) × Advisee’s risk preference (risk-avoiding vs. risk-seeking)

2.2.2 Advisors exhibited an unselective inclination to re-align with advisees’ opinions

Judgement switch ~ Baseline congruency (congruent vs. incongruent) × Advisee’s accuracy (correct vs. incorrect)

Judgement switch ~ Baseline congruency (congruent vs. incongruent) × Advisee’s confidence + Baseline confidence

OSR ~ Baseline congruency (congruent vs. incongruent) × Advisee’s accuracy (correct vs. incorrect)

OSR ~ Baseline congruency (congruent vs. incongruent) × Advisee’s accuracy (correct vs. incorrect)

+ Baseline opinion discrepancy

3.2.2 The conformity to advisees’ opinions reflects a social basis

Judgement congruency ~ Session (2 vs. 3) × Advisee condition (same vs. new)

Judgement re-alignment maintenance (in Session 3) ~ Advisee condition (same vs. new)

Self-judgement consistency (across Session 2 and 3) ~ Advisee condition (same vs. new)

4.2.1 Model-free analyses

Judgement congruency (in Session 2) ~ Preference (neutral vs. aligned vs. misaligned) × Order (neutral-aligned-misaligned vs. neutral-misaligned-aligned)

Judgement congruency (in Session 2) ~ Preference (aligned vs. misaligned)

Judgement congruency (in Session 2) ~ Trial

5.2 Results

Attention check failure ~ Study (3 vs. 4)

Investment magnitude ~ Session (1 vs. 2) × Study (3 vs. 4)

Judgement congruency (Session 2) ~ Study (3 vs. 4)

*Note.* All linear mixed-effects models reported in this table included random intercepts for both stimulus and participant, specified as + (1 | stimulus) + (1 | participant). For models that appeared multiple times in the main text, we only indicated the result section of their first occurrence here.

**Comment (6)** *STUDY 1-* *RESULTS/DISCUSSION:* *Perhaps my only major query with these findings is that (unless I misunderstood)* *the incentive structure of advice-givers is mostly rooted on advisee's performance/rewards. Namely, it could be argued that participants focus on matching advisee behavior because they do not have an immediate consequence if they are wrong. I know the incentive structure is not described fully in detail so maybe I'm missing something here, but it feels like the advisees don't really face any kind of consequence if they decide to completely disregard their own opinion and just match the advisee's. This is important not only on a higher order, but also because as it stands the trials are build in a way that advisee's judgement could be priming advice-giver responses on a lower-level processing stage (i.e. advice-givers just repeating what they just saw on screen because it is less cognitive effort).*

**Response:** We thank the reviewer for raising this concern regarding potential strategic or low-level explanations for the alignment bias and the unselective re-alignment pattern we observed in Study 1.

First, we wish to clarify the incentive structure we used for Study 1 and Study 2, and how they were communicated to the participants as below.

**2.1.3 Experimental Task** (Study 1, Lines 205-219):

“ ‘In both sessions, the advisee will first provide his/her initial opinion in each trial and then receive your advice immediately. They can decide whether to accept it as their decision in that trial (or reject it but maintaining the initial opinion). In Session 1, you are required to provide your advice to the advisee right after the display of an estate photo and a price. In Session 2, you will be additionally presented with advisee’s opinion recorded in Session 1 in each trial. After that, you provide your advice. **Before being informed of final performance in the task, the advisee would provide a subjective evaluation of your advice on a scale from 1 to 10, which was directly converted into your payment bonus (1 to 10 CNY).**’

**In fact, the subjective evaluations from advisees were randomly generated.** Notably, we explicitly informed participants that neither the advisor nor the advisee would receive performance-related feedback. This simulated real-world advice-giving contexts in which the accuracy of advice and the consequence of its adoption are not immediately verifiable. It also mitigated the possibility that alignment merely reflected an attempt to avoid evaluative penalty for misaligned that proved to be incorrect.”

**3.1.2 Experimental Task** (Study 2, Lines 511-517):

“Participants were jointly introduced to the study by the experimenter and read the cover story as we used in Study 1, including the information that they would receive the advisee’s subjective evaluation at the end of the task. **However, they were informed that their bonus would be calculated based on the trial-by-trial performance of advice (i.e., accuracy × wager, with accuracy coded as –1 for incorrect and 1 for correct).** **The overall performance score will be linearly rescaled to a 0–10 range and converted to payment bonus (1 to 10 CNY), which was added to the base payment.**”

As the reviewer correctly pointed out, participants’ incentives in Study 1 depended exclusively on the advisee’s evaluation of their advice, namely participants’ (as advisors) payments depended on how the advisees evaluated their advice. We understood the concerns that there was no immediate consequence of giving wrong advice in Study 1 from the perspective of participants, which induced a possibility that re-alignment was solely an effort-saving strategy by matching advisees’ judgements. Hence, we revised the incentive structure in Study 2, such that participants’ bonuses were directly linked to the performance of their advice, to eliminate the use of this strategy.

As this reviewer also stated in Comment #7, this re-alignment could reflect rational information use to reduce uncertainty. Moreover, this observation might also stem from an unconscious anchoring effect (or “priming” as stated by the reviewer) or opinion contagion, as we stated in the original manuscript. **These alternative interpretations share a common concern: the alignment bias does not involve social factors, but merely reflects non-social processes or unconscious influence.**

To address this possibility, we implemented a subtle yet important manipulation in a third session (Session 3) following Session 2, in Study 2. In this third session, advisees’ opinions were no longer presented, replicating the non-social condition of Session 1. Crucially, a between-subjects manipulation was introduced in Session 3: half of the participants were informed they would continue advising the same advisee from Session 2, while the other half were told they would be advising a new advisee.

This design enabled us to test whether alignment bias depends on social factors tied to the ongoing social connection. We hypothesized that if alignment bias reflected a social process, participants in the new-advisee condition would show weaker maintenance of re-alignment than those in the same-advisee condition, as no social motive persisted in this session to sustain prior re-alignment. In contrast, if alignment bias were solely driven by the low-level or unconscious processes, as suggested by the reviewer, no difference in the persistence of re-alignment would be expected between the new- and same- advisee conditions.

As we reported in the results, participants in the new-advisee condition exhibited a significantly greater decrease in judgement congruency (Session 3 – Session 2) compared to those in the same-advisee condition. Additionally, the re-aligned judgements observed in Session 2, maintained to a smaller degree in Session 3 in the new-advisee condition compared to the same-advisee condition. Importantly, our data also demonstrated that this observation could not simply be attributed to a greater motivation to maintain behavioral consistency when interacting with the same advisee.

To explicitly clarify how we ruled out these alternative interpretations and to enhance the logical progression from our rationale to the expected results, we have reorganized the following sections accordingly.

**1. Introduction** (Lines 139-143):

“In Study 2, we investigated the generalizability of this bias under a performance-based incentive structure and examined whether advisors’ re-alignment to advisees’ opinions reflected a social basis (i.e., normative conformity), or merely reflected non-social processes and unconscious influences (e.g., opinion contagion [27] or anchoring effects [28]).”

**3. Study 2** (Lines 473-476; Lines 480-488):

“However, it remained unclear whether this conformity reflected a social basis or instead arose from non-social processes (e.g., information seeking, copying strategies) and unconscious influences (e.g., opinion contagion [27] or anchoring effects [28]).”

“To disentangle alignment bias from non-social processes and unconscious influences, we introduced an additional session (Session 3) in Study 2, where participants gave advice independently again, while being informed that their advice would be provided either to the *same* advisee from Session 2 or a *new* advisee. Participants advising a new advisee were expected to exhibit a greater decline in re-alignment with the advisee’s opinions from Session 2 compared to the participants in the same-advisee condition. Conversely, if alignment bias was solely due to non-social processes or unconscious influences, no difference in the decline of re-alignment between these conditions would be anticipated.”

Additionally, we further incorporate supplemental analyses aimed at strengthening our conclusion on the social basis of the conformity to advisees’ opinions, and re-organized our results as below.

**3.1.4 Design, measurements, and analytical plans** (Lines 555-568):

“Data from Session 3 were analyzed to investigate whether the conformity to advisees’ opinions involved social motivations (i.e., normative conformity) or instead reflected non-social processes and unconscious influence. Specifically, we employed a linear mixed-effects model to test whether the decrease in judgement congruency with advisees’ Session 2 opinions, from Session 2 to Session 3, differed between participants advising the same advisee versus a new advisee. To further assess whether this difference reflected a decreased tendency to maintain re-alignment, we conducted a follow-up analysis focusing on trials where participants had previously re-aligned with the advisees’ judgements in Session 2, comparing the advisee conditions.

Additionally, the observed difference in re-alignment maintenance might alternatively reflect a greater motivation to maintain behavioral consistency when interacting with the same advisee. To examine this possibility, we further constructed a linear mixed-effects model comparing the likelihood of repeating the same judgement from Session 2 across advisee conditions.”

**3.2.2 The conformity to advisees’ opinions reflects a social basis** (Lines 611-622):

“Next, we examined the normative features of the conformity to advisees’ opinions by analyzing advice-giving patterns in Session 3. Consistent with our hypothesis, from Session 2 to Session 3, participants in the new-advisee condition exhibited a greater decrease in judgement congruency compared to those in the same-advisee condition (*β* = 0.26, *z* = 2.53, 95% CI = [0.06, 0.46], *p* = 0.01). Moreover, the re-align judgements observed in Session 2 were less likely to be maintained by participants in Session 3, in the new-advisee condition than by those in the same-advisee condition (*β* = -0.49, *z* = -2.48, 95% CI = [-0.87, -0.10], *p* = 0.01). Furthermore, this between-condition difference could not be attributed to a motivation to preserve a self-consistent image by maintaining previous judgements, as participants in the same-advisee condition did not show greater cross-session consistency in their advised judgements compared to those in the new-advisee condition (*β* = 0.20, *z* = 1.40, 95% CI = [–0.08, 0.48], *p* = 0.16).”

**3.3 Discussion** (Lines 628-633):

“Importantly, the maintenance of re-alignment with advisees’ opinions diminished when participants switched to provide advice to a new advisee, compared to the advisee they had previously interacted with. This finding ruled out the possibility that advisors’ re-alignment to advisees’ opinions merely resulted from information-seeking behaviors or having influenced by advisees’ opinions unconsciously, instead emphasizing its normative nature.”

**Comment (7)** *STUDY 1-* *RESULTS/DISCUSSION:* *You mention a negative relation between advisor's confidence in non-social judgements and judgement realignment. Please explain this further, as this may very well be evidence of advice-givers not over-relaying on advisees confidence, but not like this. Namely, if I understand correctly (but correct me if I don't), the more advisers trusted their judgement the less they changed their advise to match advisee's. But if this is the case, then it becomes somewhat important to probe how much advisees and advice-givers know about the real estate market, or even their socio-economic status. The reason why I'm saying this is because, as it stands, it could be that the overarching rule advisees are implementing is "**If I'm very confident about this one opinion I go with my own thoughts, but otherwise I default to advisees". Further testing (or at least, discussion) would be needed to rule out whether advice-givers are trying to re-align with advisees or if they are simply using them as an extra source of information to reduce their own uncertainty and realign their choices.*

**Response:** We fully understand the reviewer’s concern that the observed unselective re-alignment pattern may not necessarily reflect a social basis (normative conformity) but rather informational conformity, that advisors relied on advisees’ opinions when their own confidence is low in order to optimize their decisions.

We fully acknowledge the reviewer’s suggestion to conduct additional analyses to rule out the possibility that advisors merely followed the overarching rule described by the reviewer (‘*If I’m very confident about this one opinion I go with my own thoughts, but otherwise I default to advisees*’). We conduct supplemental analyses focusing on trials in which participants were more confidence in their own opinions then advisee did. The results showed that, advisors’ unselective re-alignment persisted, even in Study 2 where the accuracy of advice was directly incentivized. These analyses and the results are now incorporated as below.

**2.1.6 Design, measurements, and analytical plans** (Lines 357-361):

“To examine whether the likelihood of re-alignment was influenced by the accuracy of advisees’ opinions, we conducted two analyses. First, we included all trials to assess its overall effect. **Second, we focused specifically on the subset of trials where participants had indicated higher confidence in their initial judgements than their advisees did, to test whether the effect persisted.**”

**2.2.2 Advisors exhibited an unselective inclination to re-align with advisees’ opinions** (Lines 420-424):

“Moreover, in trials where participants expressed higher confidence in their initial judgements than their advisees did, the unselective tendency to re-align persisted (incorrect: *β* = 1.19, *z* = 2.57, 95% CI = [0.28, 2.10], *p* = 0.01; correct: *β* = 3.39, *z* = 6.24, 95% CI = [2.32, 4.45], *p* < 0.001).”

**3.2.1 Replications on the alignment bias and the unselective re-alignment pattern** (Lines 592-596):

“Moreover, in trials where participants expressed higher confidence in their initial judgements than their advisee did, the unselective re-alignment remained evident (incorrect: *β* = 0.70, *z* = 3.76, 95% CI = [0.34, 1.08], *p* < 0.001; correct: *β* = 1.69, *z* = 8.59, 95% CI = [1.30, 2.08], *p* < 0.001).”

Furthermore, this family of alternative explanations is further challenged by evidence from Session 3 in Study 2, as detailed in our response to Comment #6 of this reviewer. In brief, our results (“**3.2.2 The conformity to advisees’ opinions reflects a social basis**”) showed that participants in the new-advisee condition exhibited significantly weaker maintenance of re-alignment to the opinions expressed in Session 2, compared to those in the same-advisee condition. This observation could not be attribute to “*simply using them as an extra source of information to reduce their own uncertainty and realign their choice*” as stated by the reviewer.

**Comment (8)** *STUDY 2:* *I understand the rationale of this study, but my worry from the previous study compounds here: you say that participants would not persist in their realignments when facing new advisees if they acted on intention. However, if advisee's judgement is being treated as a supplementary source of information and nothing else, then advisee's being known or unknown would make no difference (which is exactly what you find in your results). Other than that I really appreciate the fact that you changed your incentive here. Good call!*

**Response:** We thank the reviewer for raising this thoughtful concern and for the positive feedback on the revised incentive structure. However, as reported in our original manuscript, we actually observed significant differences between the *same* and *new advisee* conditions, as also detailed in our response to this reviewer’s Comment #6. To facilitate better understanding of our findings and eliminate potential misinterpretation, we have re-organized this result session as below.

**3.2.2 The conformity to advisees’ opinions reflects a social basis** (Lines 611-622):

“Next, we examined the normative features of the conformity to advisees’ opinions by analyzing advice-giving patterns in Session 3. Consistent with our hypothesis, from Session 2 to Session 3, participants in the new-advisee condition exhibited a greater decrease in judgement congruency compared to those in the same-advisee condition (*β* = 0.26, *z* = 2.53, 95% CI = [0.06, 0.46], *p* = 0.01). Moreover, the re-align judgements observed in Session 2 were less likely to be maintained by participants in Session 3, in the new-advisee condition than by those in the same-advisee condition (*β* = -0.49, *z* = -2.48, 95% CI = [-0.87, -0.10], *p* = 0.01). Furthermore, this between-condition difference could not be attributed to a motivation to preserve a self-consistent image by maintaining previous judgements, as participants in the same-advisee condition did not show greater cross-session consistency in their advised judgements compared to those in the new-advisee condition (*β* = 0.20, *z* = 1.40, 95% CI = [–0.08, 0.48], *p* = 0.16).”

**Comment (9)** *STUDY 2:* *Why use a post-task survey here and not in the previous study?*

**Response:** We thank the reviewer for raising this thoughtful question. Study 2 was designed as a follow-up study to Study 1. Based on a thorough reflection after Study 1, we introduced the post-task survey in Study 2 to strengthen the manipulation check of participants’ perception of their role and the social presence of advisee. Notably, results from all post-task surveys (Studies 2–4) consistently showed that participants clearly believed their role as advisors and did not express doubts about the experimental manipulation. Given the similarity in design and implementation between Study 1 and Study 2, we believe it is reasonable to infer that participants in Study 1 also understood their advisor role and did not question the task setup. To deliver this distinction, we have incorporated a brief explanation in this section as below.

**3.1.3 Post-task survey** (Lines 529-531):

“To strengthen the manipulation check regarding participants’ perception of the interactive context (that is, a belief that they were interacting with a human advisee), a post-task survey was introduced.”

**Comment (10)** *STUDY 3-METHODS:* *The framing of the “intentionality” behind advice realignment feels somewhat overstated. It is not clearly demonstrated that participants understand the feedback as a signal for acceptance, nor that they adjust their behavior strategically in response to it. As in Study 1,* *it is still a distinct possibility that participants in the contingent condition act based on features of the feedback, and not necessarily because they are engaging in deeper intention-driven behavior.*

**Response:** We fully understand the reviewer’s concern regarding participants’ understanding of the feedback displayed from their advisees, as well as our inference of intentionality based on the feedback-driven adjustments.

First, in response to the comment that “*It is not clearly demonstrated that participants understand the feedback as a signal for acceptance*,” we recognized that we did not clearly illustrate how we induced participants’ understanding of the feedback as a signal for advisees’ acceptance on their advice.

As described in the cover story, the advisee could either accept the advice as their final decision with both judgement and wager or reject it (retaining their initial opinion). Crucially, participants were informed that neither advisor nor advisee would receive any performance feedback during the task, and their monetary bonuses depended only on actual performance (i.e., feedback from advisees was unrelated to the bonus). **This design ensured that acceptance or rejection from advisees could not be interpreted as** **performance- or reward-related cues, but rather as purely social signals of subjective approval or disapproval.** Consequently, participants perceived the feedback as socially meaningful, making subsequent behavioral adjustments more reflective of adaptive social behavior than strategic considerations. To address this concern, we have revised the manuscript to clarify that our design.

**4.1.2 Experimental Task** (Lines 664-672):

“The experiment settings were mainly consistent with Study 2, except that feedback on advice (acceptance/rejection) was disclosed in each trial after advisors submitted their advice in Session 2. According to the cover story, the advisee could either accept the advice, making it their final decision, or reject it and retain their initial opinion. Participants were explicitly informed that neither they nor the advisees had access to any information about judgement accuracy, and that their payment depended solely on their own task performance, not on the advisee’s evaluations. As a result, participants understood that the advisee’s feedback functioned purely as a social signal, rather than as an indicator of performance or monetary reward.”

Second, in response to the concern that “*it is still a distinct possibility that participants in the contingent condition act based on features of the feedback, and not necessarily because they are engaging in deeper intention-driven behavior*”, we realize that our inference of *intention* in may have overstated the level of cognitive deliberation involved in the feedback-driven advice-giving adaptations observed in Studies 3 and 4.

To address this concern, we have tempered our conclusion from the position that “acceptance-seeking intention drives alignment bias” to the more cautious position that “social feedback can shape advice-giving behavior”. This revision is grounded in the recognition that our design did not fully permit the assessment of advisors’ mental states (i.e., conscious goals) during the feedback-driven advice-giving adaptations. The relevant conclusion revisions are as below.

**4.3 Discussion** (Lines 1003-1005):

“Specifically, participants’ behaviors in response to advisees’ feedback were best explained by a reinforcement learning model, demonstrating that advice-giving tendencies can be gradually shaped by the feedback (acceptance/rejection) from advisees.”

**5.3 Discussion** (Lines 1107-1109):

“Although model-free analyses did not reveal evidence of behavioral adaptations to advisees’ feedback, model-based analyses demonstrated that advisors’ behavioral adaptations induced by advisees’ feedback persisted”

**6. General Discussion** (Lines 1126-1128; Lines 1147-1159):

“The alignment bias, characterizing advisors’ inclination to conform to advisees’ initial opinions, may be gradually shaped by feedback from advisees via a value-based learning mechanism in everyday interactions.”

“With the introduction of acceptance and rejection of advice provided by advisees, our computational modeling analyses revealed that advisors exhibited feedback-driven adaptations in their advising tendencies (to give either aligned or misaligned advice) with advisees’ fluctuating preferences. These behavioral responses to feedback were best captured by a Reinforcement Learning model. This model was grounded in an evolutionary perspective, assuming that individuals’ alignment bias may be shaped by everyday experience of advice-giving, whereby advisees exhibit a common preference for advice that aligned their initial opinions. Therefore, as advisees’ specific preferences became evident through their feedback of advice, advice-giving tendencies—providing either aligned or misaligned advice—shifted in the direction of maximizing acceptance and minimizing rejection. Moreover, even when acceptance from advisees was scant, the feedback-driven adaptations persisted, as bolstered by the model-based evidence, further suggesting that advice-giving behavior is sensitive to its consequence.”

Finally, we acknowledge that subjective reports assessing participants’ awareness of the contingency between advice type and feedback could provide more solid evidence of the intentionality behind the feedback-driven adaptations. We have added this limitation, as well as the importance of addressing it in future studies, to the General Discussion section, as below.

**6. General Discussion** (Lines 1247-1251):

“Moreover, although our design shows that advisors adjust their behavior in ways that tend to maximize acceptance and minimize rejection, it cannot determine whether this process reflects a conscious goal. Future studies could incorporate self-report-based assessment to assess whether participants deliberately attended to this feedback to guide subsequent adjustments in their advice.”

**Comment (11)** *STUDY 3-METHODS:* *The study design is clear and well-motivated, and I appreciate the addition of a pre-registered replication sample. However, the setup may not entirely support the authors' conclusion that misalignment avoidance is driven by intentional strategic behavior. Specifically, the manipulation involving advisee feedback being random or contingent is really good, but it assumes participants actively track and integrate this feedback which may not be the case. Some measure of whether participants noticed or used this contingency would greatly strengthen the argument. A post-task inference test could have addressed this issue (but I don't expect you to redo the experiment just for this!).*

**Response**: We fully acknowledge that approaches assessing participants’ awareness of their strategic use of feedback would provide more direct evidence regarding the intentional nature of alignment bias. We have added the importance of addressing it in future studies, to the General Discussion section, as below.

**6. General Discussion** (Lines 1247-1251):

“Moreover, although our design captured advisors’ behavioral adaptation that tended to maximize acceptance and minimize rejection, it could not determine whether this process reflected a conscious goal. Future studies could incorporate self-report measures to examine whether participants deliberately attended to feedback to guide subsequent advice adjustments.”

**Comment (12)** *STUDY 3-METHODS:* *Again, similar to Study 1, you refer to “mixed-effects ANOVAs,” which seems terminologically imprecise. I suggest naming them as linear mixed-effect models, and explicitly stating the structure (fixed and random effects, distributional assumptions, etc.).*

**Response**: We acknowledge the inappropriate use of the term “mixed-effects ANOVAs” and have revised the text to correctly refer to our analyses as linear mixed-effect models (see more details in the response to Comment #5).

**Comment (13)** *STUDY 3-METHODS:* *Concerning the models, please keep in mind that you may be over-parametrizing, which could in turn lead to over-fitting. Since in your approach you sort of "bake" your hypothesis into the model, and you don't really test adversarial models, some consideration on why this approach is not circular would be greatly appreciated. Some predictive use of your models or parameter recovery could help drive the point across too (I'm not requesting that you do everything, but at least something other than LOOIC)*

**Response**: We fully acknowledge the reviewer’s concern regarding the potential risk of overfitting. We confirmed that our model parameterization in the original manuscript may not be the most optimal. To address this issue and better align with our core hypothesis that “advice-giving behaviors can be shaped by advisees’ feedback of advice”, we first remove models (M8-M10) which were redundant to examine this core hypothesis.

On this basis, we have refined the structure of the remaining models (M1-M7) to avoid over-parameterization. First, due to the redundancy between *β and ω* in M2, we removed the parameter *β*, which aimed to capture participants’ pre-existing alignment tendency in the original models (in equation (6), $Q_{t}^{s}\left( align \right)= \beta$ in the original M2). We turned to not parameterizing this tendency, while replacing it as a constant (fixed as 1). Accordingly, we also conducted this refinement to M5-M7, since M5-M7 was constructed on M2.

**4.1.4 Computational modeling** (Lines 800-801; Lines 856-858):

$$\begin{aligned} Q_{t}^{s}\left( align \right)= 1\#\left( 6 \right) \end{aligned}$$

$$Q_{t}^{s}\left( misalign \right)=0$$

“Accordingly, for the initial values of $Q_{t}^{s}$, we set $Q_{t=0}^{s}\left( align \right)$ =1 and $Q_{t=0}^{s}\left( misalign \right)$ = 0, as in M2, to describe individuals’ tendencies to give aligned advice before receiving any feedback, since M2 outperformed across M1-M4.”

Second, we used separated parameters ($w_{ns}$ and $w_{s}$) to estimate the weight for non-social value and social value, and therefore refined the probability function (in equation (11)) to avoid unidentifiable parameter estimation. These revisions were as below.

**4.1.4 Computational modeling** (Lines 822-825; Line 835-843):

“Across M2 to M4, advisors determined their judgements based on the integration of non-social and social value:

$$\begin{aligned} Q_{t}=w_{ns}*Q_{t}^{s}+w_{s}*Q_{t}^{ns}\#\left( 9 \right) \end{aligned}$$

”

“Note that, in model specifications of the choice probability ($p_{t}$), we did not include the inverse Softmax temperature parameter as in M1 ($\tau$, in equation (3)). This was due to the non-social value and the social value of an option were explicitly constructed in a design-matrix fashion. Therefore, including the inverse Softmax temperature parameter would give rise to a multiplication term, which would cause unidentifiable parameter estimation [41]. The probability ($p_{t}$) of choosing an option was calculated using an inverse logit linking function:

$$\begin{aligned} p_{t}\left( higher \right)=\frac{1}{\left[ 1+e^{-\left( Q_{t}\left( higher \right)-Q_{t}\left( lower \right) \right)} \right]}\#\left( 11 \right) \end{aligned}$$

”

Using all optimized models, M7 was still the winning model. We conducted model recovery analysis across all candidate models and performed parameter recovery for the winning model (M7) to check whether all parameters could be accurately and selectively identified. The model recovery results indicated identifiability for all models, and the M7 parameter recovery results demonstrated robust recovery performance for the winning model at both the group-wise and individual-wise levels. The description of the recovery approach and the associated statistical results have been added to the supplementary materials, with references incorporated into the main text as follows.

**4.1.4 Computational modeling** (Lines 749-757):

“We further verified our winning model using two rigorous validation approaches. First, we conducted a parameter recovery analysis for the winning model to assure that all parameters could be accurately identified and recovered (**S2 Text** and **S3 Fig**). Second, as model comparison provided relative performance of the winning model, we further performed posterior predictive checks for the wining model (**S2 Text, S2 Fig**), and we found that the posterior predictions captured key behavioral patterns at the trial-wise, individual, and grand-average levels. Additionally, we conducted a full model recovery analysis to all the candidate models to ensure they were capable of the assumed behavioral patterns (**S2 Text** and **S4 Fig**).”

**Supplementary Materials (S2 Text)**:

*Parameter recovery*

Considering that there were multiple free parameters in the winning model (M7), we verified whether parameters were identifiable using parameter recovery. Denoting $\phi$ as a generic parameter for a model, we iterated the following steps:

(a) We randomly drew a set of group-level parameters from the parameter space of the winning model. That is, a group-level mean ($\mu_{\phi}$) and a group-level standard deviation ($\sigma_{\phi}$) of the parameter $\phi$. We repeated this procedure for all group-level parameters of the winning model.
(b) Next, we simulated 80 synthetic participants, whose parameters were randomly drawn from this set of group-level parameters. That is, individual-level parameters $\phi_{i}$ for 80 synthetic participants were sampled from the corresponding group-level parameters ($\mu_{\phi}$, $\sigma_{\phi}$) with a normal distribution. We repeated this procedure for all individual-level parameters of the winning model:

$$\phi_{i} \sim Normal(\mu_{\phi}, \sigma_{\phi})$$

(c) Then, we used the winning model as a generative tool to simulate behavioral data for our social advice-giving task (Session 2), namely, to simulate their advised judgement options for 66 trials (identical to our experimental task) per participant. Individuals’ judgement opinion (denoted as $\hat{D}_{i}$) were sampled from the sampling distribution conditional on individual-level parameters ($\phi_{i}$) from the previous step (i.e., likelihood function):

$$\hat{D}_{i} \sim p(\hat{D} | \phi_{i} )$$

(d) We fit the winning model to the simulated data ($\hat{D}_{i}$) in the same way as we did for the real data (D). That is, we fit the winning model to the simulated individual data ($\hat{D}_{i}$) with MCMC using Stan, and obtained parameter estimates (i.e., posterior distributions) at both the group-level (e.g., $\mu_{\phi}'$, $\sigma_{\phi}'$) and the individual level (e.g., $\phi_{i}'$).
(e) Finally, we compared whether the posterior distributions at both the group-level and the individual-level given the simulated data recovered the actual data generating parameters that were used to simulate those data, sampled from steps (a) and (b) (S3. Fig)

**Supplementary Materials (S3 Fig)**:

**S3. Fig.** Parameter recovery of the winning model. All parameters could be accurately and selectively recovered, showing proper identifiability of model parameters: (a) for the group-level, “true” parameters (in red) falling within 95% HDI of each parameter’s posterior density; (b) for the individual level, “true” parameters (x-axis) and estimated parameters (y-axis) were well correlated.

**Supplementary Materials (S2 Text)**:

*Model recovery*

We further conducted a full model-recovery test across the candidate models. This analysis aimed to clarify whether the best-fitting model is uniquely capable of explaining the data or whether alternative models could generate similar behavioral patterns. To this end, we used each candidate model to simulate 10 behavioral datasets (each dataset including 30 participants, each participant consisted of 66 trials) following the identical procedure as we conducted parameter recovery. Then, we refitted all candidate models to these simulated datasets and performed model selection procedure as we did for the real data. Successful recovery would be indicated if the model used to generate the data was also identified as the best-fitting model consistently (S4. Fig).

**Supplementary Materials (S4 Fig)**:

**S4. Fig.** Model recovery of all candidate models. Confusion matrix: *p*(fit model | simulated model) was used to illustrate the performance of model recovery. The larger value in the diagonal indicates better recovery accuracy, whereas off-diagonal values reflect potential model confusion.

**Comment (14)** *STUDY 3-RESULTS/DISCUSSION:* *The key finding—that advice-givers align more under contingent feedback—is indeed interesting, but it doesn’t necessarily imply that participants are intentionally modifying their advice to gain acceptance in a conclusive manner. The effect could also be driven by a more general reinforcement-like process (e.g., repeating previously accepted advice), without a goal-level intention to influence others. The authors should clarify whether they consider intention and reinforcement separable, and more importantly, whether they can be distinguished in this design. Overall some nuance would be appreciated.*

**Response**: As noted in our response to Comment #10, the absence of direct evidence for advisors’ subjective awareness of feedback-driven adaptations led us to temper our conclusion: rather than claiming that “acceptance-seeking intention drives alignment bias,” we now state that “social feedback can shape advice-giving behavior,” as supported by the feedback-driven adaptations observed in Studies 3 and 4. We further acknowledge this limitation in the General Discussion, where we highlight future directions that employ subjective measures to investigate the intentional basis of alignment bias.

**Comment (15)** *STUDY 3- RESULTS/DISCUSSION:* *Finally, the authors do not report (in the main text) any individual differences analyses so far. Did all participants respond similarly to the contingent vs yoked feedback? If not, understanding who adapts and why could help uncover whether the behavior is strategic or reactive.*

**Response:** We thank the reviewer for raising this valuable suggestion. We have incorporated individual data illustrations (Fig. 3b & c) and the results of descriptive analyses into the Results sections of Studies 3 and 4 to enrich our findings. Indeed, we observed an evident individual difference in participants’ feedback-driven adaptations, with 60.4% of participants in Study 3 and only 42% of participants in Study 4, indicated a larger judgement alignment rate in the phase where advisees preferred aligned advice (vs. misaligned advice).

**Fig 3. Experimental tasks in Study 3 and Study 4 and advice-giving tendencies adapted with advisees’ varying preferences.**

**4.2.1 Model-free analyses** (Lines 899-900):

“Descriptively, 60.4% (67 out of 111) of participants exhibited this direction of adaptation.”

**5.2 Results** (Lines 1070-1071):

“Descriptively, only 42% (42 out of 100) of participants exhibited this directional adaptation.”

Additionally, due to the lack of direct evidence showing the intentionality of advisors’ adaptations, we have tempered our conclusions drawn from the observation of feedback-driven adaptations.

**Comment (16)** *STUDY 4-METHODS: Beware,* *reducing advisee's acceptance ratio to 30% may serve the purpose of your manipulation but it is also rendering feedback considerably scarcer. This may have implications on the results.*

**Response:** We thank the reviewer for raising this valuable concern. We acknowledge that the manipulation of advisees’ feedback in Study 4 may have made acceptance feedback relatively scarce, potentially leading to task disengagement or low motivation—a possibility noted in this reviewer’s Comment #19 and Comment #21. We have addressed this concern in the responses to Comment #19 and Comment #21 in detail.

**Comment (17)** *STUDY 4-METHODS: Again, consider rewriting your mixed-ANOVA approach.*

**Response**: As we mentioned before, we acknowledge the inappropriate use of the term “mixed-effects ANOVAs” and have revised the text to correctly refer to our analyses as linear mixed-effect models.

**Comment (18)** *STUDY 4-METHODS: Again, do not relegate exclusions to supplementary materials.*

**Response**: We thank the reviewer for this valuable suggestion. We have now moved the OSR-related exclusion criteria from the Supplementary Material into the main text to improve readability as below.

**2.1.6 Design, measurements, and analytical plans** (Lines 336-354):

“Opinion shift rate (OSR) quantified the degree of adjustment towards advisees’ opinions. Following established measures of social influence (e.g., others’ advice [20] or opinions [34]) on individuals’ decision-making, OSR in each trial was computed as the intensity of adjustment (i.e., the distance between the opinion strength in Session 1 and the opinion strength in Session 2) divided by the baseline opinion discrepancy (i.e., the opinion discrepancy in Session 1). Specifically, an OSR larger than 0 indicates advice adjusted towards the advisee’ s opinion, while an OSR smaller than 0 indicates advice deviated from the advisee’ s opinion. For analyses involving Opinion Shift Rate (OSR) as a variable, we excluded trials involving inexplicable OSR situations: The first type of inexplicable situations occurred when the advisor’s opinion strength ($\mathrm{OS}_{t}^{\mathrm{ns}}$, indicating the advisors’ opinion strength in the non-social scenario; $\mathrm{OS}_{t}^{s}$, indicating the advisors’ opinion strength in the social scenario) shifted towards the advisee’s opinion strength ($\mathrm{OS}_{t}^{\mathrm{as}}$) (i.e., OSR > 0), however, the judgement ended up incongruent with the advisee’s judgement (e.g., $\mathrm{OS}_{t}^{\mathrm{ns}}$ = -40, $\mathrm{OS}_{t}^{\mathrm{as}}$ = -5, and $\mathrm{OS}_{t}^{s}$ = 10). This situation posed a challenge in defining whether it constituted a ‘shift towards’ or a ‘deviate from’ the advisee’s opinion. The second type of inexplicable situations occurred when $\mathrm{OS}_{t}^{\mathrm{ns}}$= $\mathrm{OS}_{t}^{\mathrm{as}}$, rendering the $\mathrm{OSR}_{t}$ value meaningless. In addition, we excluded extreme values of OSR (beyond *M* ± 3*SD* of each participant) from the analyses involving OSR as a variable.”

**Comment (19)** *STUDY 4-RESULTS/DISCUSSION: I realize as I see that indeed the models confirm the idea that there is a learning bias, that no efforts have been made to ensure these results don't come from low motivation or task disengagement.*

**Response:** We thank the reviewer for pointing out this important possibility. To address the concern that the enlarged learning bias observed in Study 4 may reflect reduced motivation or increased task disengagement, we conducted a supplemental analysis comparing participants’ failures on attention check trials in Session 2 between Study 3 and Study 4. The results showed that participants in Study 4 failed fewer attention check trials compared Study 3. This finding suggests that the learning bias observed in Study 4 was unlikely to be driven by reduced task engagement or increased distraction. The revised sections are presented below.

**5.1.3 Design, measurements, and analytical plans** (Lines 1057-1061):

“Furthermore, a linear mixed-effect model was constructed to compare task engagement between participants in Study 4 and those in Study 3, by examining the frequency of attention check failures. This analysis aimed to rule out the possibility that the absence of significant behavioral adjustments observed in Study 4 was simply driven by reduced engagement or motivation.”

**5.2 Results** (Lines 1085-1089):

“Additionally, participants in Study 4 exhibited fewer attention check failures compared to those in Study 3 (*β* = -0.26, *z* = -2.39, 95% CI = [-0.47, -0.05], *p* = 0.02), suggesting that the intensified learning bias and the lack of significant behavioral adjustments observed in Study 4 were unlikely to result from reduced task engagement.”

**Comment (20)** *STUDY 4-RESULTS/DISCUSSION: The between-study comparison meant to test the “responsibility avoidance” hypothesis—i.e., that advisors might align more in Study 3 (higher acceptance) than in Study 4 (low acceptance)—yields null results, but this comparison is underdeveloped. It’s treated as a key test but presented in a single sentence. If responsibility avoidance were a genuine driver, we would expect more behavioral alignment when the risk of influencing others is higher. But the absence of such a pattern does not prove its irrelevance; it might instead suggest the manipulation of “responsibility” (via advisee acceptance rates) is not strong or explicit enough to affect advisors’ sense of accountability A more nuanced or self-report-based assessment of responsibility perception might yield better insights.*

**Response**: We thank the reviewer for this valuable comment, which raises the important possibility that the null effect on judgement alignment observed in the comparison between Study 3 and Study 4 may reflect an ineffective manipulation of perceived advising responsibility. We acknowledge that this single result did not sufficiently rule out the possibility that alignment bias could be a result of responsibility avoiding.

Although we did not collect self-report data to directly assess whether participants in Study 4 experienced a reduced sense of responsibility compared to in Study 3, we conducted supplemental analyses examining participants’ perceived responsibility. We first compared post-survey ratings of the item “*I made my advice after careful considerations*” between participants in Study 3 and Study 4. In addition, we used investment magnitude as an indirect indicator of advising responsibility to test whether participants advised more conservatively in Study 3 than in Study 4. We have now incorporated these supplemental analyses as a post-hoc manipulation check for the perceived responsibility difference between Study 3 and Study 4, as detailed below.

**5.1.3 Design, measurements, and analytical plans** (Lines 1043-1056):

“Additionally, to further examine whether alignment bias was driven by an intention to evade advising responsibility, we first implemented a post-hoc manipulation check, examining whether participants in Study 3, where advisees accepted advice as their final decisions to an overall greater extent, experienced a stronger sense of advising responsibility compared to those in Study 4. Specifically, we first compared post-survey ratings of the item “*I made my advice after careful considerations*” between participants in Study 3 and Study 4. Behaviorally, we further confirmed this notion by investigating whether participants in Study 3 advised more cautiously, as reflected by a greater reduction in investment magnitude in Session 2 relative to Session 1 (baseline level). Based on this manipulation check, we constructed a linear mixed-effect model to compare the extent to which advisors aligned their judgements with those of advisees in Study 4 to Study 3, aiming to rule out the possibility that the alignment bias was merely driven by a desire to evade advising responsibility—an alternative account that predicts greater alignment in Study 3.”

**5.2 Results** (Lines 1093-1098):

“Compared to Study 4, advisors in Study 3 demonstrated a greater sense of responsibility in giving advice provided more conservative advice, as reflected in higher post-survey ratings of the item *“I made my advice after careful considerations”* (Mean Difference = 0.27, t(209)=2.37, 95% CI = [0.05, 0.50], *p* = 0.02), as well as a greater tendency to provide advice cautiously (measured by a greater reduction in investment relative to their baseline level) (*β* = 1.42, *t*(27394.1) = 4.46, 95% CI = [0.80, 2.05], *p* < 0.001).”

**Comment (21)** *STUDY 4-RESULTS/DISCUSSION: I worry again here that the weight of the theoretical implications of model fitting may be overstated. M7 still largely describe behavioral patterns rather than unambiguously infer psychological goals. The authors argue that persisting use of aligned advice despite evidence it won’t be accepted reflects biased feedback learning. This is plausible, but again, alternative interpretations (e.g., advice repetition as habit, default response, or even frustration-based disengagement) cannot be ruled out.*

**Response:** We thank the reviewer for pointing out this important possibility. To address the concern that the learning bias observed in Study 4 may reflect reduced motivation or increased task disengagement, we conducted a supplemental analysis comparing participants’ failures on attention check trials in Session 2 between Study 3 and Study 4. The results showed that participants in Study 4 failed fewer attention check trials compared to Study 3. This finding suggests that the learning bias observed in Study 4 was unlikely to be driven by reduced task engagement or increased distraction. The revised sections are presented below.

**5.1.3 Design, measurements, and analytical plans** (Lines 1043-1056):

“Additionally, to further examine whether alignment bias was driven by an intention to evade advising responsibility, we first implemented a post-hoc manipulation check, examining whether participants in Study 3, where advisees accepted advice as their final decisions to an overall greater extent, experienced a stronger sense of advising responsibility compared to those in Study 4. Specifically, we first compared post-survey ratings of the item “*I made my advice after careful considerations*” between participants in Study 3 and Study 4. Behaviorally, we further confirmed this notion by investigating whether participants in Study 3 advised more cautiously, as reflected by a greater reduction in investment magnitude in Session 2 relative to Session 1 (baseline level). Based on this manipulation check, we constructed a linear mixed-effect model to compare the extent to which advisors aligned their judgements with those of advisees in Study 4 to Study 3, aiming to rule out the possibility that the alignment bias was merely driven by a desire to evade advising responsibility—an alternative account that predicts greater alignment in Study 3.”

**5.2 Results** (Lines 1093-1098):

“Compared to Study 4, advisors in Study 3 demonstrated a greater sense of responsibility in giving advice provided more conservative advice, as reflected in higher post-survey ratings of the item *“I made my advice after careful considerations”* (Mean Difference = 0.27, t(209)=2.37, 95% CI = [0.05, 0.50], *p* = 0.02), as well as a greater tendency to provide advice cautiously (measured by a greater reduction in investment relative to their baseline level) (*β* = 1.42, *t*(27394.1) = 4.46, 95% CI = [0.80, 2.05], *p* < 0.001).”

**Comment (22)** *GENERAL DISCUSSION:* *It is overall principled and complete, relying adequately on the findings of all previous studies. I do insist however that this would be an adequate time to consider performance as an internal motivator to give advice, as discussed by Anllo, Hertz et al 2024 (see https://www.nature.com/articles/s44271-024-00175-5). I would also suggest that this general discussion also echoes some of the caveats and calls for caution that I mentioned above. I would encourage the authors to dial back the confidence of their conclusions or at least explicitly acknowledge these limitations.*

**Response:** We thank the reviewer for this valuable suggestion. We fully acknowledge the urgent need to revise our general discussion. Specifically, the revision of this section focusses on three aspects:

First, we conduct conceptual revision and conclusion refinements in our manuscripts. Specifically, we have tempered our conclusion from the claim that “acceptance-seeking intention drives alignment bias” to the more cautious position that “social feedback can shape advice-giving behavior.” This revision is grounded in the recognition that our design did not fully permit the assessment of advisors’ mental states (i.e., conscious goals) during these behavioral adaptations. It is therefore more appropriate to conceptualize the feedback-driven adaptation as a behavioral-level effect, offering a potential perspective on the emergence of alignment bias. The relevant conclusion revisions are as below.

**6. General Discussion** (Lines 1127-1128):

“The alignment bias, charactering advisors’ inclination to conform to advisees’ initial opinions, may be gradually shaped by feedback from advisees via a value-based learning mechanism in everyday interactions.”

“With the introduction of acceptance and rejection of advice provided by advisees, our computational modeling analyses revealed that advisors exhibited feedback-driven adaptations in their advising tendencies (to give either aligned or misaligned advice) with advisees’ fluctuating preferences. These behavioral responses to feedback were best captured by a Reinforcement Learning model. This model was grounded in an evolutionary perspective, assuming that individuals’ alignment bias may be shaped by everyday experience of advice-giving, whereby advisees exhibit a common preference for advice that aligned their initial opinions. Therefore, as advisees’ specific preferences became evident through their feedback of advice, advice-giving tendencies—providing either aligned or misaligned advice—shifted in the direction of maximizing acceptance and minimizing rejection. Moreover, even when acceptance from advisees was scant, the feedback-driven adaptations persisted, as bolstered by the model-based evidence, further suggesting that advice-giving behavior is sensitive to its consequence.”

Additionally, to align with our revised interpretation of the results concerning the presence-dependent maintenance of re-alignment in Study 2, we have updated the corresponding conclusion as follows (Lines 1138-1141).

“Crucially, our data showed that this conformity could not be rather attributed to over-reliance on irrelevant information of judgement accuracy or low-level processes (e.g., information seeking, response copying, or unconscious susceptibility to others’ opinions).”

Second, we conduct refinements of the discussion on theoretical implications. As suggested by the reviewer, we incorporated the idea of performance as an internal motivator in advice-giving, as discussed by Anllo, Hertz et al. (2024). This perspective offers a valuable theoretical foundation for our study and closely aligns with our findings on the multifaceted nature of advice-giving tendencies. We believe that integrating this viewpoint deepens our theoretical interpretation and enhances the contribution of our work. Specifically, we incorporated this relevant work into the discussion of the theoretical implications as follows (Lines 1197-1214).

“A recent study provides further insight into the variability of individuals’ willingness to offer opinions as advice [25]. Individuals tend to share their opinions as advice when they are more confident in their performance. However, individuals are also more likely to advise when informed that their advice would be presented to future participants with their performance ratings, indicating that advice-giving behavior is not only shaped by perceived performance but also by social incentives such as reputational rewards. In line with previous findings showing that individuals modulate their confidence to enhance social influence, these results further support the view that, beyond the internal motivation to offer accurate advice, advice-giving behavior is also underlain a desire to manage reputation. On the other hand, research on social exclusion offers another perspective: the act of securing acceptance may also stem from an aversion to rejection. Social rejection is inherently aversive, as it signals threats to one’s sense of belonging and self-worth [55, 56]. As demonstrated by research on normative conformity, the need to belong even often overrides the pursuit of accuracy [13, 34, 57]. Taken together, these findings—highlighting both the desire for social rewards and the avoidance of social punishment—provide a deeper understanding of the motivational basis underlying advice-giving behaviors, and further elucidate why social outcomes may ultimately introduce systematic bias and compromise the accuracy of advice.”

Third, we incorporated additional evidence from newly conducted analyses in response to the reviewers’ previous concerns. These findings provide critical support for our conclusions by helping to rule out alternative explanations for the observed results. The newly included analyses are summarized below (Lines 1141-1146).

“This was evidenced in a post-interaction session, where re-alignment with advisees’ opinions was maintained to a reduced degree once their interaction ended, compared to when participants were told they would continue interacting with the same advisee. Importantly, this difference could not be explained by a tendency to maintain self-consistency (by repeating previous judgements) in the presence of the same advisee.”

Finally, to address the reviewer’s concerns raised in earlier comments—particularly those that could not be fully resolved by the current design and data—we have added further discussion on the limitations of our experimental implementation and the generalizability of our conclusions, as outlined below (Lines 1247-1251).

“Moreover, although our design shows that advisors adjust their behavior in ways that tend to maximize acceptance and minimize rejection, it cannot determine whether this process reflects a conscious goal. Future studies could incorporate self-report-based assessment to assess whether participants deliberately attended to this feedback to guide subsequent adjustments in their advice.”

**Reviewer #3:**

*This manuscript presents a novel and important perspective on "alignment bias" in advice-giving behavior, emphasizing that advisors may not only influence advisees but also be influenced by them in return. This view of reverse social influence enriches our understanding of social interaction mechanisms and, to our knowledge, represents the first systematic computational modeling of such dynamics in the advice-giving literature. The experimental design is rigorous and the logic across the four progressive studies is coherent. The authors build and validate the cognitive mechanism of alignment bias with clarity. However, the manuscript can be further improved in the following respects.*

**Response**: We thank the Reviewer for their positive feedback on our research. We have fully considered all comments and suggestions and address each of them here below.

**Comment (1)** *On computational modeling: The* *authors have not conducted parameter recovery prior to model fitting. This is strongly recommended to ensure that the model can reliably recover the underlying parameters, especially given the observed correlation between* *β and ω parameters, which raises concerns about potential parameter redundancy. Additionally, model recovery should be conducted to assess whether the models are distinguishable based on key behavioral patterns. Moreover, while the authors use hierarchical Bayesian modeling to estimate parameters of the RL models, they do not report posterior distributions, credible intervals, or MCMC diagnostics (e.g., R-hat or Gelman–Rubin statistics). Providing parameter distribution plots and convergence diagnostics would enhance the transparency and reliability of the model estimation.*

**Response:** We fully acknowledge the reviewer’s concern regarding the potential risk of parameter redundancy. We confirmed that our model parameterization in the original manuscript indeed suffered from over-parameterization (especially between *β and ω*) after conducting parameter recovery. To address this issue and better align with our core hypothesis that “advice-giving behaviors can be shaped by advisees’ feedback of advice”, we first remove models (M8-M10) which were redundant to examine this core hypothesis.

On this basis, we have refined the structure of the remaining models (M1-M7) to avoid over-parameterization. First, due to the redundancy between *β and ω* in M2, we removed the parameter *β*, which aimed to capture participants’ pre-existing alignment tendency in the original models (in equation (6), $Q_{t}^{s}\left( align \right)= \beta$ in the original M2). We turned to not parameterizing this tendency, while replacing it as a constant (fixed as 1).

**4.1.4 Computational modeling** (Lines 800-801; Lines 856-858):

$$\begin{aligned} Q_{t}^{s}\left( align \right)= 1\#\left( 6 \right) \end{aligned}$$

$$Q_{t}^{s}\left( misalign \right)=0$$

“Accordingly, for the initial values of $Q_{t}^{s}$, we set $Q_{t=0}^{s}\left( align \right)$ =1 and $Q_{t=0}^{s}\left( misalign \right)$ = 0, as in M2, to describe individuals’ tendencies to give aligned advice before receiving any feedback, since M2 outperformed across M1-M4.”

Second, we used separated parameters ($w_{ns}$ and $w_{s}$) to estimate the weight for non-social value and social value, and therefore refined the probability function (in equation (11)) to avoid unidentifiable parameter estimation. These revisions were as below.

**4.1.4 Computational modeling** (Lines 822-825; Line 835-843):

“Across M2 to M4, advisors determined their judgements based on the integration of non-social and social value:

$$\begin{aligned} Q_{t}=w_{ns}*Q_{t}^{s}+w_{s}*Q_{t}^{ns}\#\left( 9 \right) \end{aligned}$$

”

“Note that, in model specifications of the choice probability ($p_{t}$), we did not include the inverse Softmax temperature parameter as in M1 ($\tau$, in equation (3)). This was due to the non-social value and the social value of an option were explicitly constructed in a design-matrix fashion. Therefore, including the inverse Softmax temperature parameter would give rise to a multiplication term, which would cause unidentifiable parameter estimation [41]. The probability ($p_{t}$) of choosing an option was calculated using an inverse logit linking function:

$$\begin{aligned} p_{t}\left( higher \right)=\frac{1}{\left[ 1+e^{-\left( Q_{t}\left( higher \right)-Q_{t}\left( lower \right) \right)} \right]}\#\left( 11 \right) \end{aligned}$$

”

Using all optimized models, M7 was still the winning model. We conducted model recovery analysis across all candidate models and performed parameter recovery for the winning model (M7) to check whether all parameters could be accurately and selectively identified. The model recovery results indicated identifiability for all models, and the M7 parameter recovery results demonstrated robust recovery performance for the winning model at both the group-wise and individual-wise levels. The description of the recovery approach and the associated statistical results have been added to the supplementary materials, with references incorporated into the main text as follows.

**4.1.4 Computational modeling** (Lines 749-757):

“We further verified our winning model using two rigorous validation approaches. First, we conducted a parameter recovery analysis for the winning model to assure that all parameters could be accurately identified and recovered (**S2 Text** and **S3 Fig**). Second, as model comparison provided relative performance of the winning model, we further performed posterior predictive checks for the wining model (**S2 Text, S2 Fig**), and we found that the posterior predictions captured key behavioral patterns at the trial-wise, individual, and grand-average levels. Additionally, we conducted a full model recovery analysis to all the candidate models to ensure they were capable of the assumed behavioral patterns (**S2 Text** and **S4 Fig**).”

**Supplementary Materials (S2 Text)**:

*Parameter recovery*

Considering that there were multiple free parameters in the winning model (M7), we verified whether parameters were identifiable using parameter recovery. Denoting $\phi$ as a generic parameter for a model, we iterated the following steps:

(a) We randomly drew a set of group-level parameters from the parameter space of the winning model. That is, a group-level mean ($\mu_{\phi}$) and a group-level standard deviation ($\sigma_{\phi}$) of the parameter $\phi$. We repeated this procedure for all group-level parameters of the winning model.
(b) Next, we simulated 80 synthetic participants, whose parameters were randomly drawn from this set of group-level parameters. That is, individual-level parameters $\phi_{i}$ for 80 synthetic participants were sampled from the corresponding group-level parameters ($\mu_{\phi}$, $\sigma_{\phi}$) with a normal distribution. We repeated this procedure for all individual-level parameters of the winning model:

$$\phi_{i} \sim Normal(\mu_{\phi}, \sigma_{\phi})$$

(c) Then, we used the winning model as a generative tool to simulate behavioral data for our social advice-giving task (Session 2), namely, to simulate their advised judgement options for 66 trials (identical to our experimental task) per participant. Individuals’ judgement opinion (denoted as $\hat{D}_{i}$) were sampled from the sampling distribution conditional on individual-level parameters ($\phi_{i}$) from the previous step (i.e., likelihood function):

$$\hat{D}_{i} \sim p(\hat{D} | \phi_{i} )$$

(d) We fit the winning model to the simulated data ($\hat{D}_{i}$) in the same way as we did for the real data (D). That is, we fit the winning model to the simulated individual data ($\hat{D}_{i}$) with MCMC using Stan, and obtained parameter estimates (i.e., posterior distributions) at both the group-level (e.g., $\mu_{\phi}'$, $\sigma_{\phi}'$) and the individual level (e.g., $\phi_{i}'$).
(e) Finally, we compared whether the posterior distributions at both the group-level and the individual-level given the simulated data recovered the actual data generating parameters that were used to simulate those data, sampled from steps (a) and (b) (S3. Fig)

**Supplementary Materials (S3 Fig)**:

**S3. Fig.** Parameter recovery of the winning model. All parameters could be accurately and selectively recovered, showing proper identifiability of model parameters: (a) for the group-level, “true” parameters (in red) falling within 95% HDI of each parameter’s posterior density; (b) for the individual level, “true” parameters (x-axis) and estimated parameters (y-axis) were well correlated.

**Supplementary Materials (S2 Text)**:

*Model recovery*

We further conducted a full model-recovery test across the candidate models. This analysis aimed to clarify whether the best-fitting model is uniquely capable of explaining the data or whether alternative models could generate similar behavioral patterns. To this end, we used each candidate model to simulate 10 behavioral datasets (each dataset including 30 participants, each participant consisted of 66 trials) following the identical procedure as we conducted parameter recovery. Then, we refitted all candidate models to these simulated datasets and performed model selection procedure as we did for the real data. Successful recovery would be indicated if the model used to generate the data was also identified as the best-fitting model consistently (S4. Fig).

**Supplementary Materials (S4 Fig)**:

**S4. Fig.** Model recovery of all candidate models. Confusion matrix: *p*(fit model | simulated model) was used to illustrate the performance of model recovery. The larger value in the diagonal indicates better recovery accuracy, whereas off-diagonal values reflect potential model confusion.

Furthermore, we have added the convergence diagnostics (R-hats) and parameter posterior distribution plots to the supplementary materials as below.

**S1 Table. The largest R̂ values of all parameters of the winning model.**

|  | $w_{s}$ | $w_{ns}$ | $a_{al}^{ac}$ | $a_{al}^{re}$ | $a_{mis}^{ac}$ | $a_{mis}^{re}$ |
| --- | --- | --- | --- | --- | --- | --- |
| Study 3 | 1.009 | 1.011 | 1.012 | 1.008 | 1.010 | 1.015 |
| Study 4 | 1.010 | 1.006 | 1.007 | 1.011 | 1.014 | 1.003 |

*Note.* R̂ values of all parameters were close to 1.0 (smaller than 1.1 at most in the current study), which indicated adequate convergence.

**S2 Fig. Posterior predictive check of the winning model and the posterior distributions of all estimated parameters.** (a) and (e) Trial-wise posterior predicative check. The actual trial-by-trial data well correlated by the predicted data. (b) and (f) Grand-wise posterior predicative check. The true data (dark green vertical line) fall within the 95% Highest Density Intervals (HDIs) of each posterior density (in light green). (c) and (g) Individual-wise posterior predicative check. The actual individual data well correlated by the predicted data. (d) and (h) The posterior distributions of the estimated parameter in the winning model. The vertical line indicates the posterior median, and the blue shading represents the 95% HDI.

**Comment (2)** *Theoretical interpretation of acceptance-seeking: The authors interpret alignment bias as being driven by an acceptance-seeking motivation. However, in everyday situations (e.g., choosing a restaurant or making a consumer decision) which is mentioned in the manuscript as something to be simulated, where advisors and advisees share similar expertise, accuracy may not be critical. The manuscript should further elaborate on the psychological meaning of acceptance-seeking: why are individuals motivated to seek acceptance at the cost of advice accuracy? What is the underlying psychological or social reward structure that justifies this behavior?*

**Response:** We appreciate the reviewer’s insightful question regarding the psychological basis of acceptance-seeking and why individuals might prioritize it over advice accuracy in certain contexts. To address this concern, we have added a more elaborated discussion in the General Discussion section of the revised manuscript, clarifying the psychological significance and empirical basis of this motivational drive as below.

**6. General Discussion** (Lines 1197-1214)**:**

“A recent study provides further insight into the variability of individuals’ willingness to offer opinions as advice [25]. Individuals tend to share their opinions as advice when they are more confident in their performance. However, individuals are also more likely to advise when informed that their advice would be presented to future participants with their performance ratings, indicating that advice-giving behavior is not only shaped by perceived performance but also by social incentives such as reputational rewards. In line with previous findings showing that individuals modulate their confidence to enhance social influence, these results further support the view that, beyond the internal motivation to offer accurate advice, advice-giving behavior is also underlain a desire to manage reputation. On the other hand, research on social exclusion offers another perspective: the act of securing acceptance may also stem from an aversion to rejection. Social rejection is inherently aversive, as it signals threats to one’s sense of belonging and self-worth [55, 56]. As demonstrated by research on normative conformity, the need to belong even often overrides the pursuit of accuracy [13, 34, 57]. Taken together, these findings—highlighting both the desire for social rewards and the avoidance of social punishment—provide a deeper understanding of the motivational basis underlying advice-giving behaviors, and further elucidate why social outcomes may ultimately introduce systematic bias and compromise the accuracy of advice.”

**Comment (3)** *Insufficient exclusion of alternative explanations: The current account focuses on acceptance-seeking but does not rule out other plausible motives such as rejection aversion. It is also possible that advisors treat advisees’ initial opinions as informational cues under uncertainty rather than being socially influenced. Future studies could include control conditions where the source of the initial opinion is non-social (e.g., AI agents or historical data) to distinguish social motives from cue integration strategies.*

**Response:** We sincerely thank the reviewer for raising this important point. We address the concern in three parts.

First, in the current study, we conceptualize acceptance and rejection as psychologically opposing feedback of advice giving. In other words, we integrated rejection aversion/avoiding as a component of the broader feedback-driven effect. In accordance with this conceptualization, in the reinforcement model we implemented, advisees’ acceptance was modeled as reward (Outcome = 1) that reinforced the advising tendency, while advisees’ rejection was modeled as punishment (Outcome = 0) that reduced the likelihood of maintaining the advising tendency.

Notably, we have tempered our conclusion from the claim that “acceptance-seeking intention drives alignment bias” to the more cautious position that “social feedback can shape advice-giving behavior.” This revision is grounded in the recognition that our design did not fully permit the assessment of advisors’ mental states (i.e., intentions) during these behavioral adaptations. It is therefore more appropriate to conceptualize the feedback-driven adaptation as a behavioral-level effect, which could serve as a potential perspective on the emergence of alignment bias. The relevant conclusion revisions are as below.

**6. General Discussion** (Lines 1126-1128; Lines 1147-1159):

“The alignment bias, characterizing advisors’ inclination to conform to advisees’ initial opinions, may be gradually shaped by feedback from advisees via a value-based learning mechanism in everyday interactions.”

“With the introduction of acceptance and rejection of advice provided by advisees, our computational modeling analyses revealed that advisors exhibited feedback-driven adaptations in their advising tendencies (to give either aligned or misaligned advice) with advisees’ fluctuating preferences. These behavioral responses to feedback were best captured by a Reinforcement Learning model. This model was grounded in an evolutionary perspective, assuming that individuals’ alignment bias may be shaped by everyday experience of advice-giving, whereby advisees exhibit a common preference for advice that aligned their initial opinions. Therefore, as advisees’ specific preferences became evident through their feedback of advice, advice-giving tendencies—providing either aligned or misaligned advice—shifted in the direction of maximizing acceptance and minimizing rejection. Moreover, even when acceptance from advisees was scant, the feedback-driven adaptations persisted, as bolstered by the model-based evidence, further suggesting that advice-giving behavior is sensitive to its consequence.”

Second, we fully acknowledge the reviewer’s concern on the possibility that advisors treat advisees’ opinions as informational cues. To address this possibility, in Study 2, we implemented a subtle manipulation in a third session following Session 2 (where advisees’ opinions were fully disclosed). In this third session, advisees’ opinions were no longer presented, replicating the non-social condition of Session 1. Crucially, a between-subjects manipulation was introduced: half of the participants were informed they would continue advising the same advisee from Session 2, while the other half were told they would be advising a new advisee.

This design enabled us to test whether alignment bias depends on social factors tied to the ongoing social connection. We hypothesized that if alignment bias reflected a social process, participants in the new-advisee condition would show weaker maintenance of re-alignment than those in the same-advisee condition, as no social motive persisted in this session to sustain prior re-alignment. In contrast, if alignment bias were solely driven by the low-level or unconscious processes, as suggested by the reviewer, no difference in the persistence of re-alignment would be expected between the new- and same- advisee conditions.

As we reported in the results, participants in the new-advisee condition exhibited a significantly greater decrease in judgement congruency (Session 3 – Session 2) compared to those in the same-advisee condition. Additionally, the re-aligned judgements observed in Session 2, maintained to a smaller degree in Session 3 in the new-advisee condition compared to the same-advisee condition. Importantly, our data also demonstrated that this observation could not simply be attributed to a greater motivation to maintain behavioral consistency when interacting with the same advisee.

To explicitly clarify how we ruled out these alternative interpretations and to enhance the logical progression from our rationale to the expected results, we have reorganized the following sections accordingly.

**1. Introduction** (Lines 139-143):

“In Study 2, we investigated the generalizability of this bias under a performance-based incentive structure and examined whether advisors’ re-alignment to advisees’ opinions reflected a social basis (i.e., normative conformity), or merely reflected non-social processes and unconscious influences (e.g., opinion contagion [27] or anchoring effects [28]).”

**3. Study 2** (Lines 473-476; Lines 480-488):

“However, it remained unclear whether this conformity reflected a social basis or instead arose from non-social processes (e.g., information seeking, copying strategies) and unconscious influences (e.g., opinion contagion [27] or anchoring effects [28]).”

“To disentangle alignment bias from non-social processes and unconscious influences, we introduced an additional session (Session 3) in Study 2, where participants gave advice independently again, while being informed that their advice would be provided either to the *same* advisee from Session 2 or a *new* advisee. Participants advising a new advisee were expected to exhibit a greater decline in re-alignment with the advisee’s opinions from Session 2 compared to the participants in the same-advisee condition. Conversely, if alignment bias was solely due to non-social processes or unconscious influences, no difference in the decline of re-alignment between these conditions would be anticipated.”

Additionally, we further incorporate supplemental analyses aimed at strengthening our conclusion on the social basis of the conformity to advisees’ opinions, and re-organized our results as below.

**3.1.4 Design, measurements, and analytical plans** (Lines 555-568):

“Data from Session 3 were analyzed to investigate whether the conformity to advisees’ opinions involved social motivations (i.e., normative conformity) or instead reflected non-social processes and unconscious influence. Specifically, we employed a linear mixed-effects model to test whether the decrease in judgement congruency with advisees’ Session 2 opinions, from Session 2 to Session 3, differed between participants advising the same advisee versus a new advisee. To further assess whether this difference reflected a decreased tendency to maintain re-alignment, we conducted a follow-up analysis focusing on trials where participants had previously re-aligned with the advisees’ judgements in Session 2, comparing the advisee conditions.

Additionally, the observed difference in re-alignment maintenance might alternatively reflect a greater motivation to maintain behavioral consistency when interacting with the same advisee. To examine this possibility, we further constructed a linear mixed-effects model comparing the likelihood of repeating the same judgement from Session 2 across advisee conditions.”

**3.2.2 The conformity to advisees’ opinions reflects a social basis** (Lines 611-622):

“Next, we examined the normative features of the conformity to advisees’ opinions by analyzing advice-giving patterns in Session 3. Consistent with our hypothesis, from Session 2 to Session 3, participants in the new-advisee condition exhibited a greater decrease in judgement congruency compared to those in the same-advisee condition (*β* = 0.26, *z* = 2.53, 95% CI = [0.06, 0.46], *p* = 0.01). Moreover, the re-align judgements observed in Session 2 were less likely to be maintained by participants in Session 3, in the new-advisee condition than by those in the same-advisee condition (*β* = -0.49, *z* = -2.48, 95% CI = [-0.87, -0.10], *p* = 0.01). Furthermore, this between-condition difference could not be attributed to a motivation to preserve a self-consistent image by maintaining previous judgements, as participants in the same-advisee condition did not show greater cross-session consistency in their advised judgements compared to those in the new-advisee condition (*β* = 0.20, *z* = 1.40, 95% CI = [–0.08, 0.48], *p* = 0.16).”

**3.3 Discussion** (Lines 628-633):

“Importantly, the maintenance of re-alignment with advisees’ opinions diminished when participants switched to provide advice to a new advisee, compared to the advisee they had previously interacted with. This finding ruled out the possibility that advisors’ re-alignment to advisees’ opinions merely resulted from information-seeking behaviors or having influenced by advisees’ opinions unconsciously, instead emphasizing its normative nature.”

Fourth, we fully acknowledge the reviewer’s valuable suggestion to incorporate non-social sources into design (e.g., AI agents or historical data) in future research. We have now included this as a future direction in the General Discussion section as below (Lines 1261-1264).

“Future studies could further probe the social complexity of alignment bias by comparing advisors’ responses to human advisees versus non-social targets (e.g., AI), to examine whether the observed unselective re-alignment patterns differ between these contexts.”

**Comment (4)** *Missing experimental details: Some details regarding the experimental procedures—such as the exact number of trials per condition—are missing. This hinders reproducibility and should be clearly reported.*

**Response:** We thank the reviewer for pointing out this important issue. In the revised manuscript, we have now provided all missing details regarding the experimental procedures, including the exact number of trials per condition, in the Methods sections of each study as below.

**2.1.3 Experimental Task** (Study 1, Lines 225-226; Lines 229-233):

“In summary, participants were tasked with providing advice in both *non-social* and *social* scenarios sequentially (**Fig 1a**), each consisting of 66 trials using identical stimuli.”

“In the social scenario (Session 2), participants observed all the stimuli again and provided advice in an interactive scenario, after the interacting advisee proposed their initial opinions (both judgements and investments) on the stimuli, except for 20% of trials (14 trials) where advisees’ opinions were masked (identical to the *non-social* scenario).”

**3.1.2 Experimental Task** (Study 2, Lines 518-521):

“Additionally, in Study 2, the task comprised 3 sessions. Session 1 and 2 were consistent with Study 1. In Session 3, which included the same number of trials and identical stimuli as Sessions 1 and 2, participants again provided advice independently as in Session 1.”

**4.1.2 Experimental Task** (Study 3, Lines 674-675):

“Session 2 was truncated into three phases, each consisting of 22 trials, and advisees’ preferences fluctuated between phases (**Fig 3a**).”

In addition, we have incorporated the background settings and the details of the cover story shown to the participants, as well as the details of the post-task survey as below.

**2.1.3 Experimental Task** (Study 1, Lines 192-219):

“Before the experiment, two participants entered the lab at the same time and received the study instructions together. In this verbal instruction, they were informed that each participant would be assigned either the role of advisor or advisee, to convince them that they were indeed playing a real-time interaction game. The advisee’s bonus depends directly on the number of wagers won through their decisions, with the advisor’s role being to assist the advisee in maximizing winnings by providing advice. Both players completed two sessions involving identical stimuli, with the later session serving a second chance to revise decisions or advice (this was designed to conceal the true purpose of the manipulation). During the game, neither players would receive feedback on their accuracy or performance. After this verbal introduction, participants entered separate booths and received role-specific instructions. In fact, all participants were assigned to the advisor role and read the cover story as below.

‘In both sessions, the advisee will first provide his/her initial opinion in each trail and then receive your advice immediately. They can decide whether to accept it as their decision in that trial (or reject it but maintaining the initial opinion). In Session 1, you are required to provide your advice to the advisee right after the display of an estate photo and a price. In Session 2, you will be additionally presented with advisee’s opinion recorded in Session 1 in each trial. After that, you provide your advice. Before being informed of final performance in the task, the advisee would provide a subjective evaluation of your advice on a scale from 1 to 10, which was directly converted into your payment bonus (1 to 10 CNY).’

In fact, the subjective evaluations from advisees were randomly generated. Notably, we explicitly informed participants that neither the advisor nor the advisee would receive performance-related feedback. This simulated real-world advice-giving contexts in which the accuracy of advice and the consequence of its adoption are not immediately verifiable. It also mitigated the possibility that alignment merely reflected an attempt to avoid evaluative penalty for misaligned that proved to be incorrect.”

**3.1.2 Experimental Task** (Study 2, Lines 508-517):

“Different from Study 1, four participants entered the lab at the same time, to make them believe that there were two advisor-advisee pairs (hence two potential advisees). This was designed to enable the manipulation regarding a “new” advisee in Session 3. Participants were jointly introduced to the study by the experimenter and read the cover story as we used in Study 1, including the information that they would receive the advisee’s subjective evaluation at the end of the task. However, they were informed that their bonus would be calculated based on the trial-by-trial performance of advice (i.e., accuracy × wager, with accuracy coded as –1 for incorrect and 1 for correct). The overall performance score will be linearly rescaled to a 0–10 range and converted to payment bonus (1 to 10 CNY), which was added to the base payment.”

**3.1.3 Post-task survey** (Study 2, Lines 543-550):

“Second, upon completion of the experiments, participants were instructed to share their subjective experiences during the experiments, in response to experimenter’s open-end asking, ‘Did you have any thoughts or impressions about the advisee player you were interacting with during the task?’. Notably, Participants were not informed in advance that they would be asked this question, and we made no indication that their responses would have any influence, in order to minimize response bias and encourage honest answers. Those who indicated that they perceived the advisees as fictitious would also be excluded.”

**Comment (5)** *Overall, the manuscript addresses an important and underexplored question with strong methodological foundations. With further clarification on the computational modeling and refinement of the theoretical framing, the study will make a valuable contribution to both social psychology and computational modeling literature.*

**Response**: We sincerely thank the Reviewer for their positive feedback on our research.

**Reviewer #4:**

*The study is carefully executed, and its findings will interest scholars across disciplines, from behavioural science to evolutionary ecology—for instance, in understanding the evolution of teaching behaviour, which is rare in the animal kingdom.*

**Response**: We sincerely thank the Reviewer for their positive feedback on our research.

**Comment (1)** *Functional implications: As someone interested in the evolutionary game dynamics of human social-learning strategies, I would welcome a discussion of the game-theoretic consequences of advice-alignment bias. If advisers merely echo advisees’ opinions, what adaptive value remains for the advisees in seeking advice at all? Elaborating on the functional rationale and downstream consequences of alignment would enrich the manuscript.*

**Response**: We sincerely thank the Reviewer for their insightful comment and their interest in the broader functional implications of our findings. Below, we expand the discussion to address the evolutionary and game-theoretic consequences of advice-alignment bias.

**6. General Discussion** (Lines 1224-1233):

“However, from a game-theoretic perspective, when advisees express their opinions before seeking advice, the situation creates a functional dilemma that exposes an inherent conflict between advisors’ social interests and the epistemic utility of advice for advisees. In other words, while advisors may pursue alignment to gain approval and satisfaction from advisees, such aligned advice may simultaneously lose epistemic value and be underappreciated. This tension underscores the need for future research to conceptualize advice-giving as a deliberative trade-off between epistemic accuracy and reputational or relational gains. Moreover, identifying when and how advisees recognize and adjust for biased alignment could offer valuable insight into the reciprocal dynamics that characterize multi-round social interactions.”

**Comment (2)** *Model-recovery analysis: The RL modelling is compelling, and it is reassuring that the calibrated model reproduces human behaviour. However, the manuscript would be stronger with a full model-recovery test across the candidate models. This would clarify whether the best-fitting model is uniquely capable of explaining the data or whether alternative models can generate similar behavioural patterns.*

**Response**: We sincerely thank the Reviewer for the acknowledgement of the RL modeling. Based on the refinement of models, we performed model recovery analyses across all candidate models, and conducted parameter recovery for the winning model (M7) to check whether all parameters could be accurately and selectively identified. The model recovery results indicated identifiability for all models, and the parameter recovery results demonstrated robust recovery performance for the winning model at both the group-wise and individual-wise levels. The description of the recovery approach and the associated statistical results have been added to the supplementary materials, with references incorporated into the main text as follows.

**4.1.4 Computational modeling** (Lines 749-757):

“We further verified our winning model using two rigorous validation approaches. First, we conducted a parameter recovery analysis for the winning model to assure that all parameters could be accurately identified and recovered (**S2 Text** and **S3 Fig**). Second, as model comparison provided relative performance of the winning model, we further performed posterior predictive checks for the wining model (**S2 Text, S2 Fig**), and we found that the posterior predictions captured key behavioral patterns at the trial-wise, individual, and grand-average levels. Additionally, we conducted a full model recovery analysis to all the candidate models to ensure they were capable of the assumed behavioral patterns (**S2 Text** and **S4 Fig**).”

**Supplementary Materials (S2 Text)**:

*Parameter recovery*

Considering that there were multiple free parameters in the winning model (M7), we verified whether parameters were identifiable using parameter recovery. Denoting $\phi$ as a generic parameter for a model, we iterated the following steps:

(a) We randomly drew a set of group-level parameters from the parameter space of the winning model. That is, a group-level mean ($\mu_{\phi}$) and a group-level standard deviation ($\sigma_{\phi}$) of the parameter $\phi$. We repeated this procedure for all group-level parameters of the winning model.
(b) Next, we simulated 80 synthetic participants, whose parameters were randomly drawn from this set of group-level parameters. That is, individual-level parameters $\phi_{i}$ for 80 synthetic participants were sampled from the corresponding group-level parameters ($\mu_{\phi}$, $\sigma_{\phi}$) with a normal distribution. We repeated this procedure for all individual-level parameters of the winning model:

$$\phi_{i} \sim Normal(\mu_{\phi}, \sigma_{\phi})$$

(c) Then, we used the winning model as a generative tool to simulate behavioral data for our social advice-giving task (Session 2), namely, to simulate their advised judgement options for 66 trials (identical to our experimental task) per participant. Individuals’ judgement opinion (denoted as $\hat{D}_{i}$) were sampled from the sampling distribution conditional on individual-level parameters ($\phi_{i}$) from the previous step (i.e., likelihood function):

$$\hat{D}_{i} \sim p(\hat{D} | \phi_{i} )$$

(d) We fit the winning model to the simulated data ($\hat{D}_{i}$) in the same way as we did for the real data (D). That is, we fit the winning model to the simulated individual data ($\hat{D}_{i}$) with MCMC using Stan, and obtained parameter estimates (i.e., posterior distributions) at both the group-level (e.g., $\mu_{\phi}'$, $\sigma_{\phi}'$) and the individual level (e.g., $\phi_{i}'$).
(e) Finally, we compared whether the posterior distributions at both the group-level and the individual-level given the simulated data recovered the actual data generating parameters that were used to simulate those data, sampled from steps (a) and (b) (S3. Fig)

**Supplementary Materials (S3 Fig)**:

**S3. Fig.** Parameter recovery of the winning model. All parameters could be accurately and selectively recovered, showing proper identifiability of model parameters: (a) for the group-level, “true” parameters (in red) falling within 95% HDI of each parameter’s posterior density; (b) for the individual level, “true” parameters (x-axis) and estimated parameters (y-axis) were well correlated.

**Supplementary Materials (S2 Text)**:

*Model recovery*

We further conducted a full model-recovery test across the candidate models. This analysis aimed to clarify whether the best-fitting model is uniquely capable of explaining the data or whether alternative models could generate similar behavioral patterns. To this end, we used each candidate model to simulate 10 behavioral datasets (each dataset including 30 participants, each participant consisted of 66 trials) following the identical procedure as we conducted parameter recovery. Then, we refitted all candidate models to these simulated datasets and performed model selection procedure as we did for the real data. Successful recovery would be indicated if the model used to generate the data was also identified as the best-fitting model consistently (S4. Fig).”

**Supplementary Materials (S4 Fig)**:

**S4. Fig.** Model recovery of all candidate models. Confusion matrix: *p*(fit model | simulated model) was used to illustrate the performance of model recovery. The larger value in the diagonal indicates better recovery accuracy, whereas off-diagonal values reflect potential model confusion.
