# Supplementary material for: Do we advise as one likes? The alignment bias in social advice giving
Source: PLoS Comput Biol. 2025 Dec 2;21(12):e1013732. doi: 10.1371/journal.pcbi.1013732 (PMC12688123; doi:10.1371/journal.pcbi.1013732)
Supplement: Attachment — Submitted filename: Response_to_Reviewers_auresp_2.docx [file pcbi.1013732.s007.docx]

**Dear Prof. Zhiyi Chen**

Thank you for allowing us to revise and re-submit our manuscript “Do we advise as one likes? The alignment bias in social advice giving” (**PCOMPBIOL-D-25-00735**). We want to express our sincere appreciation to you and the reviewers for all your perceptive remarks and valuable recommendations on our manuscript. We hope the reviewers find that we have addressed each of the comments and that you agree that our revised manuscript has significantly improved. We attach the manuscript with all changes indicated in blue. Below, we provide responses to each of the reviewers’ comments.

**Reviewer #1:** *The authors took reviewer concerns seriously and made numerous revisions. I very much appreciated the added context for the social aspects of the manipulation and methodological details provided throughout.*

**Response**: We thank the Reviewer for their positive feedback on our research. We have fully considered all comments and suggestions and address each of them here below.

**Comment (1):** *Some points of clarification:*

- ***Title:*** *"Do we advise as we like? The alignment bias in social advice giving" -- the mix of plural 'we' and singular 'one' in the original is confusing, because it implies that 'one' is somehow not part of the 'we'.*

**Response**: We appreciate the reviewer’s comment. We would like to clarify that the title is intentionally *“Do we advise as one likes? The alignment bias in social advice giving.”* The use of “one” was to convey a general and impersonal meaning (i.e., “Do we advise as other people like or prefer?”). We apologize for any confusion caused by the phrasing.

- ***Line 134****: "and who would provide a subjective evaluation of advice after interaction" -- should be "their advice" or even something like "their performance in the task" as the roles and experimental structure are only made clear later.*
- **Response**: We have revised the sentence to read “and who would provide a subjective evaluation of their advice after interaction,” which improves clarity at this stage of the manuscript.
- ***Line 219****: "an attempt to avoid evaluative penalty for misaligned that proved to be incorrect." missing word -- probably this is "misaligned advice."*
- **Response**: We have corrected the phrase to “an attempt to avoid evaluative penalty for misaligned advice that proved to be incorrect.”
- ***Line 549****: "Those who indicated that they perceived the advisees as fictious would also be excluded." -- Verb tense introduces doubt / seems like speculation. Were any participants excluded due to this criteria?*

**Response**: We thank the reviewer for pointing this out. We have revised the sentence to:
“Participants who indicated that they perceived the advisees as fictitious would have been excluded; however, no participants met this criterion, and therefore none were excluded on this basis.” (now Line 551)

- ***Line 1197, 1247, 1264, perhaps elsewhere:*** *Given the size of this paragraph, it may be reasonable to make a new paragraph here to aid the reader.*

**Response**: We have divided these long paragraphs into shorter ones to improve readability and aid the reader’s comprehension.

- ***Line 1209****: Missing '.'*

**Response**: The missing period has been added.

**Reviewer #2:** *I thank the authors for the careful review work they conducted, I have nothing to add. I believe the manuscript improved its quality and I'm happy with its current state.*

**Reviewer #3:** *The authors have solved all my previous questions and the quality of the manuscript has been greatly improved. I have no further concerns.*

**Reviewer #4:** *The authors have responded to all the comments I made, as well as posted by other reviewers thoroughly, and their responses were compelling. I appreciate the authors' effort in conducting new simulations and analysis for the model recovery and parameter recovery.*

**Response**: We sincerely thank Reviewer #2, Reviewer #3, and Reviewer #4 for their positive and constructive feedback. We greatly appreciate the time and effort they invested in reviewing our manuscript.
